# Pleiotropy facilitates local adaptation to distant optima in common ragweed (*Ambrosia artemisiifolia*)

**DOI:** 10.1371/journal.pgen.1008707

**Published:** 2020-03-25

**Authors:** Tuomas Hämälä, Amanda J. Gorton, David A. Moeller, Peter Tiffin

**Affiliations:** 1 Department of Plant and Microbial Biology, University of Minnesota, St. Paul, Minnesota, United States of America; 2 Department of Ecology, Evolution and Behavior, University of Minnesota, St. Paul, Minnesota, United States of America; University of Cologne, GERMANY

## Abstract

Pleiotropy, the control of multiple phenotypes by a single locus, is expected to slow the rate of adaptation by increasing the chance that beneficial alleles also have deleterious effects. However, a prediction arising from classical theory of quantitative trait evolution states that pleiotropic alleles may have a selective advantage when phenotypes are distant from their selective optima. We examine the role of pleiotropy in regulating adaptive differentiation among populations of common ragweed (*Ambrosia artemisiifolia*); a species that has recently expanded its North American range due to human-mediated habitat change. We employ a phenotype-free approach by using connectivity in gene networks as a proxy for pleiotropy. First, we identify loci bearing footprints of local adaptation, and then use genotype-expression mapping and co-expression networks to infer the connectivity of the genes. Our results indicate that the putatively adaptive loci are highly pleiotropic, as they are more likely than expected to affect the expression of other genes, and they reside in central positions within the gene networks. We propose that the conditionally advantageous alleles at these loci avoid the cost of pleiotropy by having large phenotypic effects that are beneficial when populations are far from their selective optima. We further use evolutionary simulations to show that these patterns are in agreement with a model where populations face novel selective pressures, as expected during a range expansion. Overall, our results suggest that highly connected genes may be targets of positive selection during environmental change, even though they likely experience strong purifying selection in stable selective environments.

## Author summary

Theoretical studies examining the genetic basis of adaptation often predict that loci controlling multiple traits are under strong negative selection, because they have an increased chance of deleterious effects. However, such loci also tend to have large effects on phenotypes, which might be beneficial when populations are adapting to new environments. We test this hypothesis by using a widely-distributed annual plant, common ragweed (*Ambrosia artemisiifolia*), as our study species. Climate change after the last ice-age and the spread of agriculture has led ragweed to expand its North American range, exposing populations to novel environment stressors. We use genetic variants and gene expression data to infer how likely are loci involved in climate adaptation to control multiple traits. Our results show that loci bearing signatures of local adaptation are situated in central positions within gene networks, from where they affect the expression of many other genes. This high connectivity likely means that these adaptive loci also affect multiple phenotypes. We therefore present an empirical case where adaptation to new environments has resulted in loci controlling multiple phenotypes to be subject to positive selection, even though the same loci would likely be under negative selection in stable environments.

## Introduction

Early theory by Fisher [1] suggests that adaptation mainly advances through the fixation of small-effect loci, because pleiotropy (the control of multiple phenotypes by a single locus) would cause large effect loci to move phenotypes away from their fitness optima. Since then, multiple authors have concluded that pleiotropy can constrain the rate of adaptation by increasing the probability that even if mutations have beneficial effects on some traits they might have deleterious effects on others [2–5]. An extension of the Fisher’s “geometric model” has, however, shown that the effect sizes of loci fixed by positive selection are proportional to the distance from the trait optima [6]. When populations adapt to new environments, phenotypes are initially far from their optimal values. This gives a selective advantage to loci with large phenotypic effects, as they can move phenotypes faster towards the optima. By contrast, when phenotypes are nearing their optimal values, large-effect loci become less beneficial because they can overshoot the optimum [6]. Experimental data indicates that the effect-sizes of loci increase with increasing level of pleiotropy [7–9], suggesting that highly pleiotropic loci may escape the “cost of complexity” [2] and be selectively advantageous if phenotypes are distant from their selective optima.

The role of pleiotropy in local adaptation has mainly been studied with genetic mapping [10–15]. For example, studies applying quantitative trait loci (QTL) mapping on multiple traits have often found overlapping QTL regions, suggestive of pleiotropy [10,12,15,16]. However, disentangling the true signals of pleiotropy from artifacts caused by linked markers or correlated phenotypes is challenging [17–19]. As genetic mapping is further restricted to finding associations between genotypes and a set of preselected traits, alternative methods may hold promise to provide novel insights into the role of pleiotropy in local adaptation. One potential approach for quantifying pleiotropic effects is to use molecular networks to study the connectivity between loci, as more highly connected genes are likely to be more pleiotropic [20–23]. Such networks can be based on protein-protein interactions, metabolic pathways, or gene regulatory interactions [24]. In line with the Fisher’s theory, multiple studies have found evidence that genes with greater connectivity are under stronger selective constraint [25–32], and that targets of long-term positive selection tend to be found at the periphery of gene networks [33–35]. However, despite the general observation that more highly connected genes evolve at a slower rate, studies examining biochemical pathways in yeast and humans have found that more central genes may, in fact, be responsible for short-term responses to selection [35,36]. These results raise questions about the adaptive potential of network connectivity, and whether connectivity helps or hinders the evolution of local adaptation.

In this study, we investigate the relationship between local adaptation and network connectivity using transcriptome data collected from a widely-distributed wind-pollinated plant, *Ambrosia artemisiifolia* (common ragweed). *A*. *artemisiifolia* started colonizing new environments across North America as a consequence of post-glacial climate change, but the rate of this range expansion has recently increased due to human-mediated habitat change [37–39]. Populations are locally adapted to these new environments despite low genetic structure and high gene flow across much of the species’ range [38–42]. We identify loci that contribute to local adaptation using genetic variants called from transcriptome data collected across a wide geographical range. We then perform two sets of analyses to infer the relationship between expression connectivity and the loci bearing signatures of local adaptation. First, we search for associations between genetic variants and gene expression levels to assess how likely are the putatively adaptive genes to affect the expression of other genes. We then construct a co-expression network and use it to analyze how the adaptive loci are distributed within network modules. We find that the adaptive loci are more likely than non-adaptive loci to affect the expression of other genes, and that these loci are in more central positions in co-expression networks than is expected by chance. Last, using evolutionary simulations, we show that the observation of greater pleiotropy among loci bearing signatures of differential selection is in agreement with a model in which populations are adapting to novel environments. Taken together, our results are consistent with a positive relationship between pleiotropy and recent adaptive differentiation.

## Results

**Fig 1:**
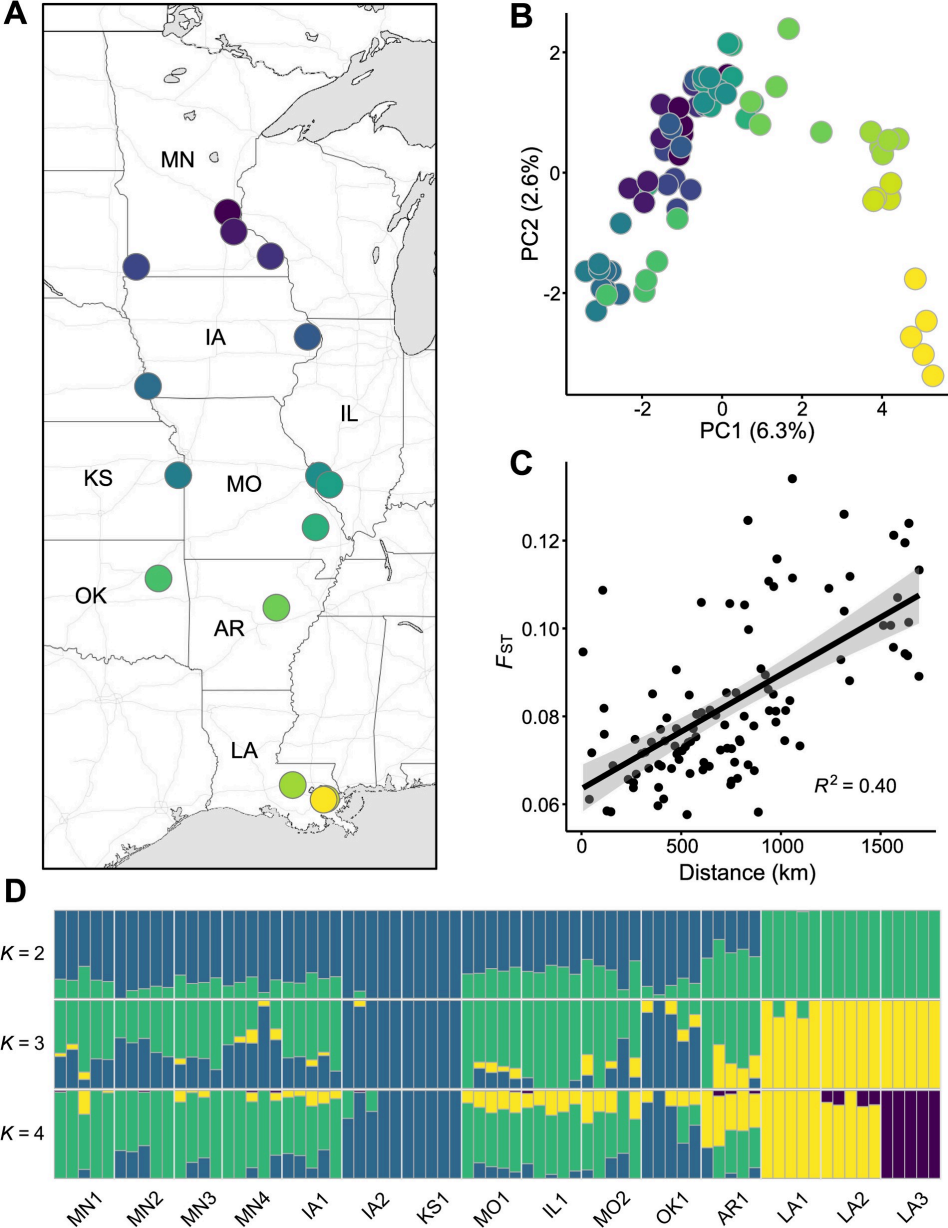
Population structure in *A*. *artemisiifolia*. **A**: Map showing locations of the populations. Map tiles by Stamen Design, under CC BY 3.0. **B**: Genetic variation along the first two axes of a principal components analysis (PCA). Variation explained by the PCs is shown in brackets. **C**: The increase of genetic distance (*F*_ST_) as a function of geographical distance between the populations. **D**: Estimated admixture proportions for three different numbers of ancestral populations (*K*).

We collected transcriptome data from 74 *A*. *artemisiifolia* individuals, sampled from 15 geographically-defined populations (Fig 1A). Our sampling covers 15 degrees of latitude and 7 degrees of longitude, thereby capturing considerable variation in environmental factors such as temperature and precipitation (S1 Fig). The sequencing yielded a total of 1448 M reads, with an average of 19.5 M reads per individual. We first assembled a de novo transcriptome to function as a reference for alignments. BUSCO [43] analysis indicated that the 58,644 contigs contained 85% of the screened near-universal single-copy orthologs (4% fragmented and 11% missing). On average, 93% (from 89% to 97% per individual) of all reads aligned successfully to this assembly. After filtering out contigs with low expression, 38,111 contigs were used for the variant calling. The variant calling produced an initial 7.4 M SNPs and indels, out of which 650,580 biallelic SNPs passed the filtering steps based on mapping quality, read coverage, minor allele frequency, heterozygosity, and missing data. These SNPs were located in 18,927 individual contigs (hereafter referred to as genes).

Although de novo transcriptomes are a useful tool for studying genetics in non-model species [44], assembly errors combined with the difficulty of assessing build-quality of the transcriptome might bias the downstream analyses. To evaluate whether these complications influence our results, we also identified transcribed genes using a reference genome of a closely related species, *Helianthus annuus* (Asteraceae, common sunflower) [45]. Approximately 60% of *A*. *artemisiifolia* reads aligned uniquely to the *H*. *annuus* genome, indicating that sequence-conservation at genic regions is relatively high between the two species. Despite a considerable difference in the number of genes which varied at the sequence- and expression-levels (18,927 for the *A*. *artemisiifolia* transcriptome and 8933 for the *H*. *annuus* reference genome), analyses of the two datasets yielded similar results. Because the results are similar, and the putatively novel transcripts that do not align to *H*. *annuus* reference genome may be important for local adaptation, results reported in the main text are based on the de novo transcriptome data.

### High genetic diversity and low population structure

Estimators of the population mutation rate (θ = 4*N*_*e*_*μ*) indicated that *A*. *artemisiifolia* harbors high levels of genetic diversity: θ_π_ = 0.02 and θ_W_ = 0.03. Tajima’s *D* was strongly negative, *D* = –1.57, likely indicating a recent population size increase [46]. Consistent with previous reports [38,39], our *F*_ST_ and principal components analysis (PCA) revealed relatively little differentiation among the 15 populations (S1 Table and Fig 1B; see S2 Fig for PCs with the *H*. *annuus* reference-aligned reads), and population-specific diversity estimates were similar across populations (S2 Table). The only exception were three populations from Louisiana (LA) that formed a distinct cluster along the second axis of the PCA plot. We also observed a pattern of isolation-by-distance, as the between population *F*_ST_ estimates increased with geographical distance (Fig 1C), and population positions along PC1 were related to a north-south gradient (Fig 1B). An admixture analysis conducted with PCAngsd [47] suggested that the most likely number of ancestral populations (*K*) is two, corresponding to a northern cluster and a southern cluster, the latter of which consisted largely of individuals from Louisiana. Populations from more central latitudes exhibited some admixture between the two genetic groups (Fig 1D and S3 Fig). We also examined the division of individuals at *K* = 3 and *K* = 4. The genetic differentiation of individuals from Louisiana was further emphasized by these results: at *K* = 3 the Louisiana individuals formed a genetic cluster that was distinct from the other samples, and at *K* = 4, one population from Louisiana was further isolated (Fig 1D and S3 Fig).

### Selection scan reveals spatially varying selection

We used two conceptually different approaches to find loci potentially contributing to local adaptation: LFMM 2 [48], a method based on genotype-environment correlations; and PCAdapt [49], an environmentally naïve method based on among-population differentiation in allele frequencies. Studies conducted on simulated data have found that these methods generally perform well under different demographic scenarios, and that they compare favorably against other methods of similar design [50,51]. Population structure in the LFMM analysis was controlled by including *K* = 4 latent factors in the model [48]. We defined main axes of environmental variation using a PCA based on 19 BIOCLIM variables [52]. We used the first four PCs, which explained 97.5% of the total variance (S3 Table), as predictors in the LFMM models. Out of 650,580 SNPs, we discovered 4700 SNPs, distributed across 2038 genes, harboring signals of selection in response to environmental variation summarized by the PCs (*q* < 0.05). 2724 SNPs were associated with PC1, 128 with PC2, 14 with PC3, and 1834 with PC4. The PCAdapt analysis identified 3190 SNPs, representing 2103 unique genes, showing higher than expected associations with the genotype-PC axes (*K* = 4). Of these SNPs, 1054 were associated with PC1, 340 with PC2, 1608 with PC3, and 188 with PC4. Approximately 28% of the genes were identified by both methods (*P* < 2 × 10^−16^, Fisher’s exact test), suggesting that despite a significant overlap these methods are not completely redundant.

We focus most of our subsequent analyses on genes housing the outlier SNPs, as they present the best candidates for adaptive loci. However, because these genes have different lengths, ones with large number of SNPs may have increased chance of being affected by false positives. To evaluate whether the number of segregating sites (*S*) found within genes increases the probability of a gene being identified as a target of local adaptation, we examined the relationship between *S* within each gene and the lowest observed *P-*values from the LFMM and PCAdapt models. Among all genes in the transcriptome, *P-*values were negatively correlated with *S* ($R_{\mathrm{LFMM}}^{2}$ = 0.13, $R_{\mathrm{PCAdapt}}^{2}$ = 0.13). However, a linear regression model (*P*-values normalized to mean of zero and standard deviation of one as the response, and *S* and the outlier status of the genes as predictors) revealed that *P*-values of the putatively adaptive loci differed strongly from the non-candidate loci even after controlling for *S* (*β*_LFMM_ = –2.11, *β*_PCAdapt_ = –2.20, *P* < 2 × 10^−16^), indicating that the outlier status of a gene is robust to the number segregating sites.

### Differentiation outliers bear signatures of selective sweeps

**Fig 2:**
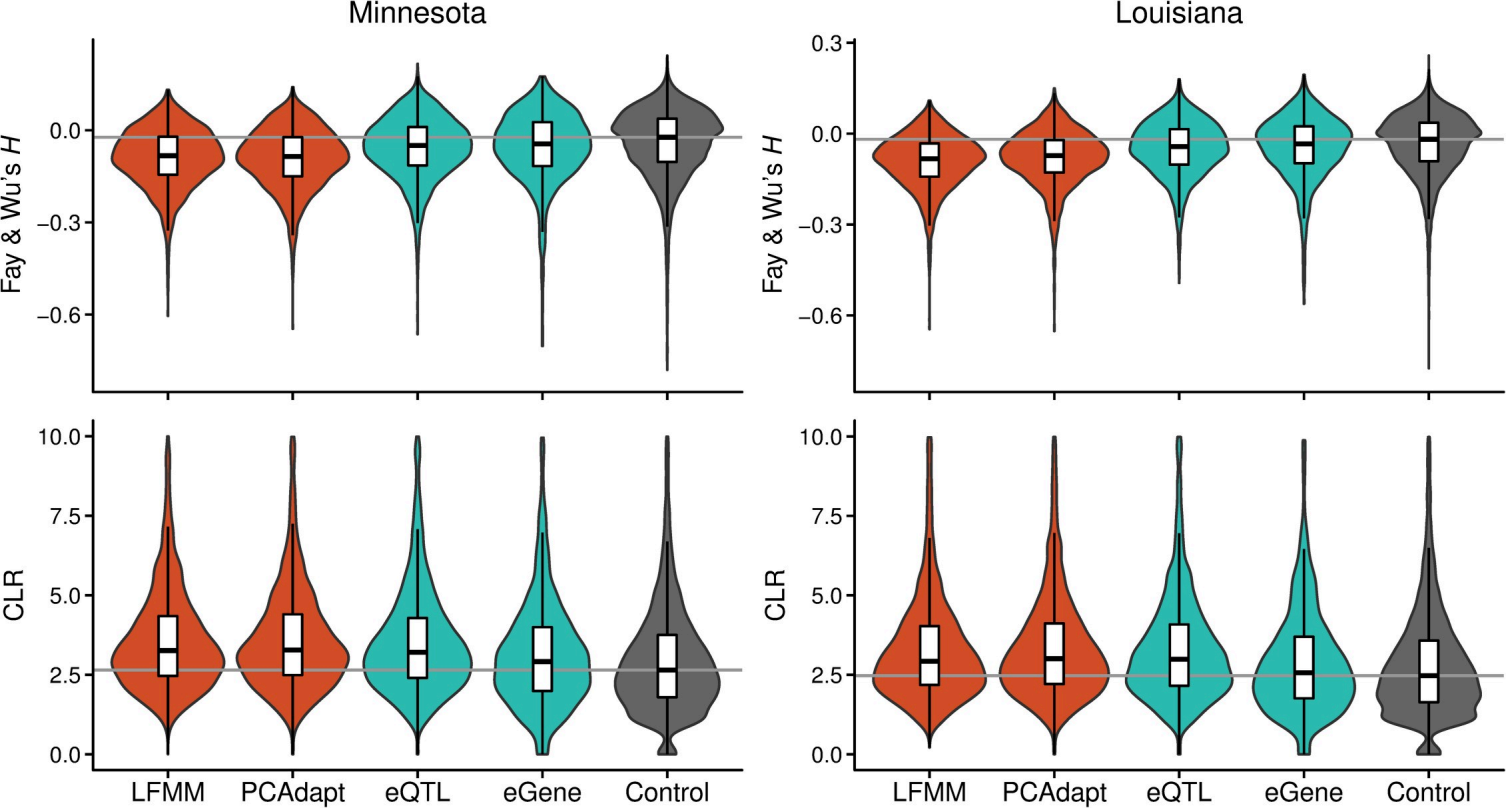
Evidence of selective sweeps at candidate genes. Fay and Wu’s *H* and CLR distributions of the selection outliers, eQTLs, and eGenes are compared against the rest of the transcriptome for two datasets. The horizontal lines mark the medians of the control genes.

We then examined the sequence variation of the outlier genes to assess whether they carry additional signatures of recent selection. We conducted this analysis separately for individuals from the northern and southern edges of our sampling distribution, Minnesota (MN, *n* = 19) and Louisiana (LA, *n* = 15). Environment driven selection is expected to have the greatest effect on allele frequencies at these locations (S1 Fig), although more fine-scale differences in the selective environment (related to e.g. pathogens [53]) likely exist among the study populations. Indeed, genetic differentiation between MN and LA was considerably higher for the outlier than the non-outlier loci (S4 Fig; *P* < 2 × 10^−16^, Wilcoxon rank-sum test). However, as drift can easily inflate population differentiation, we used additional statistics to search for footprints of positive selection. Measures of population mutation rate, θ_π_ and θ_W_, revealed that outliers among both datasets harbored potential signals of hitchhiking by having lower diversity than other genes in the transcriptome (S5 Fig; *P* < 2 × 10^−16^, Wilcoxon rank-sum test) [54]. To examine this possibility in more detail, we estimated a neutrality measure, Fay and Wu’s *H* [55], for the genes. In both populations, the outlier genes had *H* estimates that were more negative than expected based on all genes (Fig 2; *P* < 2 × 10^−16^, Wilcoxon rank-sum test), suggesting localized enrichments of derived variants due to positive selection [55]. We then estimated composite likelihood ratios (CLR) [56,57] to further assess the probability that positive selection has skewed the frequencies of segregating sites at the outlier genes. The CLR estimates strengthened the evidence for selective sweeps by showing clear deviations between the outlier and non-outlier genes (Fig 2; *P* < 2 × 10^−16^, Wilcoxon rank-sum test). All these statistics are consistent with positive selection having acted on the outlier genes, with signals slightly stronger on genes identified by both LFMM and PCAdapt (S6 Fig). To evaluate whether *H* or CLR statistics can be biased by the outlier loci having more segregating sites, on average, than the non-outlier loci, we conducted regression-based permutation tests. First, we fitted the following linear models to the MN and LA datasets: *H* or CLR = *S* + outlier status. We then randomized the outlier assignment across genes and compared the fit of the randomized model against the observed model. Out of 10,000 repeats, < 5% of the random models exceeded the fit of the observed models. Taken together, these results provide evidence that allele frequency differentiation at the outlier genes has been driven by recent positive selection.

### eQTLs are overrepresented and eGenes underrepresented among the differentiation outliers

**Fig 3:**
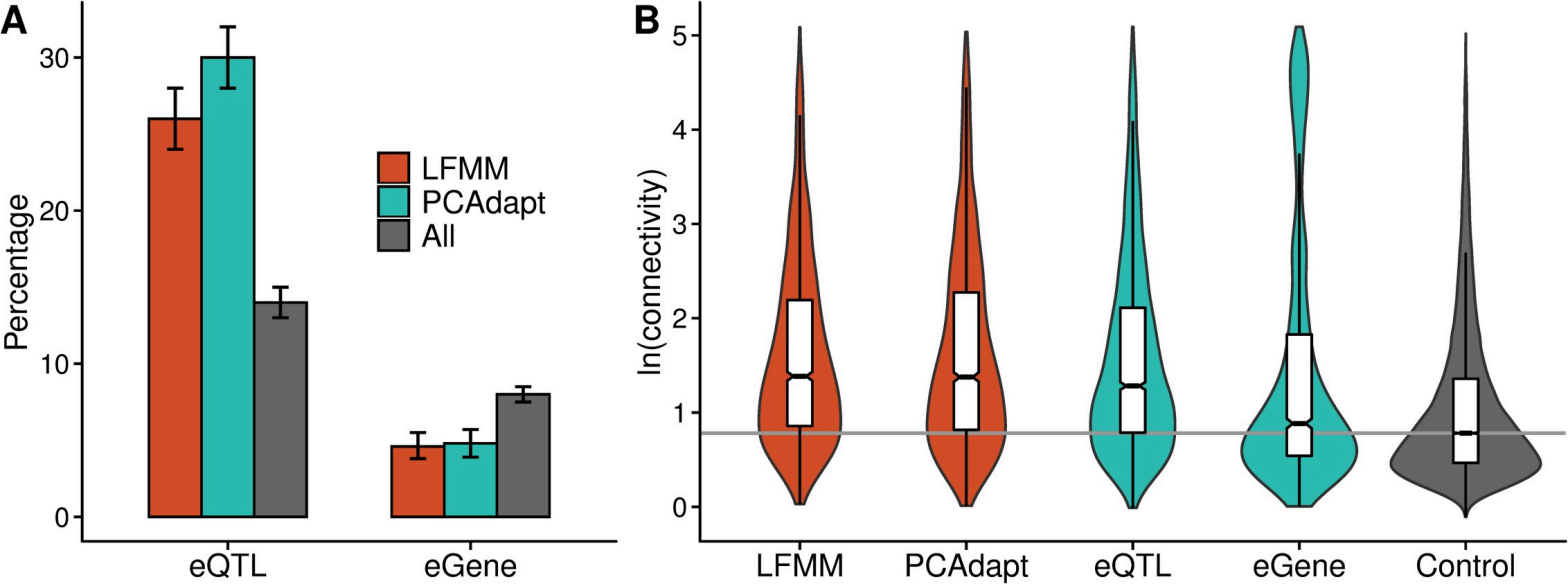
Gene network topology at the candidate genes. **A**: The percentage of eQTLs and eGenes found among the LFMM and PCAdapt outliers compared against all genes. Error bars show 95% bootstrap-based CIs. **B**: Connectivity measures at candidate genes. The horizontal line marks the median of the control genes.

To evaluate whether local adaptation outliers are more pleiotropic than expected, we searched for associations between SNPs and gene expression to identify which of the genes contain *trans*-regulatory variants associated with the expression of other genes. Out of 38,111 genes, 18,927 had variable sites and sufficient expression level to be included into expression QTL (eQTL) mapping. Population structure was controlled by removing the effects of two main principal components from the expression data (see Methods) and by including admixture proportions (*K* = 4) as cofactors in the model. A total of 2676 (14%) genes carried SNPs showing genotype-expression associations (hereafter referred to as eQTL) at a nominal *q*-value threshold of 0.05. These eQTLs were associated with the expression of 1537 (8%) genes, which we refer to as eGenes. 186 (1%) genes were identified as both eQTL and eGene. Among the 2038 outlier genes identified by LFMM, 520 (26%) were eQTLs, whereas 626 out of 2103 (30%) PCAdapt outliers were eQTLs. Compared to the transcriptome-wide background, both outlier sets harbored more eQTLs than expect by chance (*P* < 2 × 10^−16^, Fisher’s exact test). This pattern remained after controlling for the number of segregating sites (logistic regression: *β*_LFMM_ = 0.50, *β*_PCAdapt_ = 0.79, *P* < 2 × 10^−16^). In contrast, only 95 (5%) of the LFMM outliers and 101 (5%) of the PCAdapt outliers were eGenes; fewer than expected by chance (*P* < 4.0 × 10^−10^, Fisher’s exact test) (Fig 3A, see S7A Fig for the *H*. *annuus* reference-aligned reads). We note, however, that the number of eGenes is likely an underestimate, as our mapping cannot discover genes associated with *cis*-eQTLs that reside in intergenic regions. Next, we examined whether the number of eGenes controlled by an eQTL differs between the selection-outliers and the transcriptome-wide background. Consistent with previous reports on expression-mapping [18], most eQTLs had an association only with a single eGene (median = 1, mean = 1.6), leading to a highly skewed distribution. We therefore modelled the data as a binomial distribution (single eGene = 0, more than one eGenes = 1). A logistic regression with *S* as a cofactor indicated that the outlier-eQTLs were associated with the expression of multiple eGenes more often than non-outliers (*β*_LFMM_ = 0.31, *β*_PCAdapt_ = 0.43, *P* < 0.003), indicative of outlier eQTLs having greater pleiotropic affects than non-outlier eQTLs.

### Differentiation outliers are highly connected

To further assess the functional importance of outlier loci, we examined the positions of those genes in a co-expression network. The co-expression network consisted of 31 modules, with individual modules containing between 72 and 8781 genes (median of 373 genes). Eight modules harbored more LFMM and PCAdapt outliers than expected by chance *(q* < 0.05, Fisher’s exact test), potentially representing gene-groups associated with physiological or developmental processes linked to local adaptation [58].

**Fig 4:**
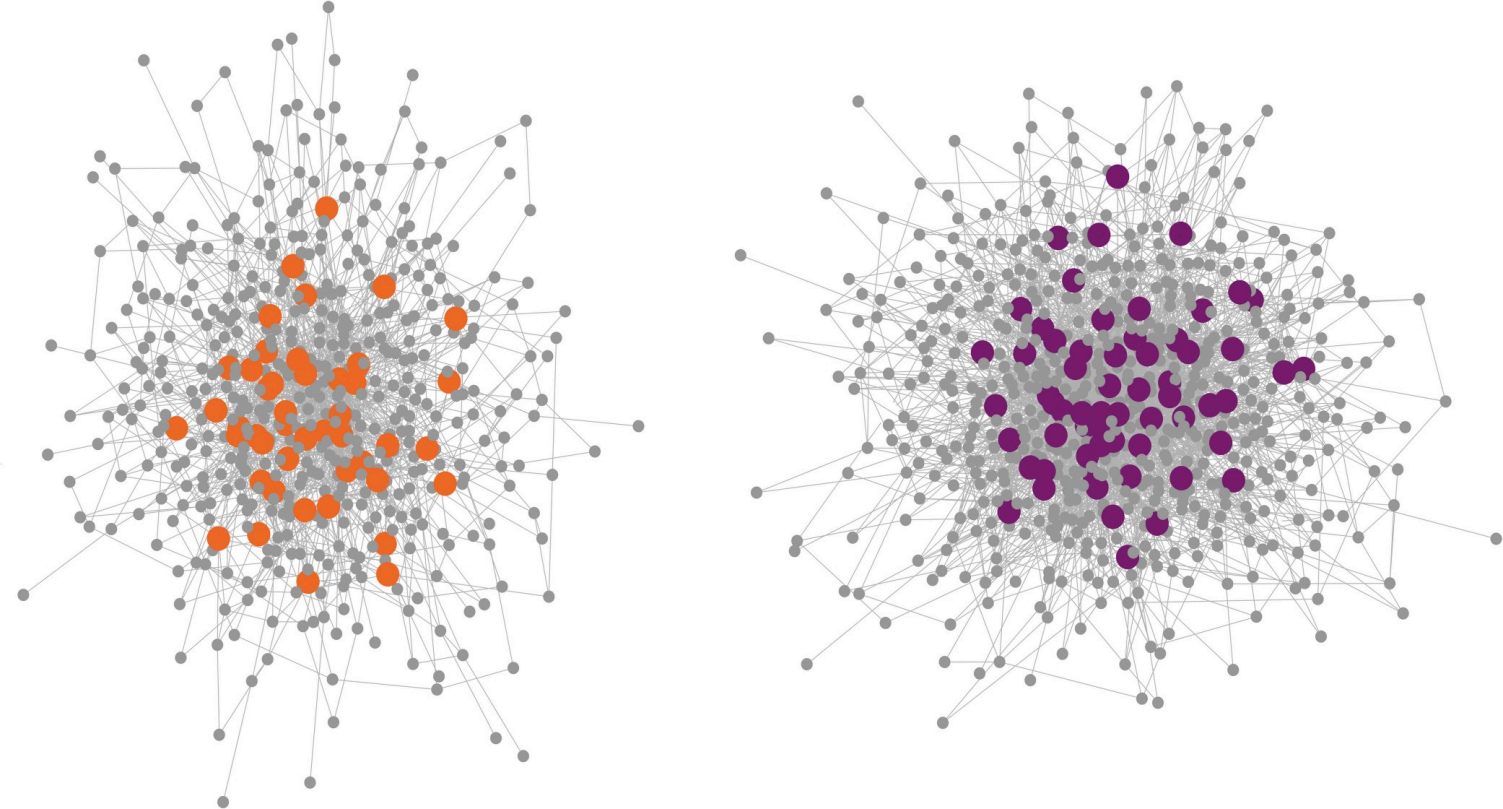
Visual representation of two network modules containing more than expected LFMM and PCAdapt outliers. Circles represent genes and lines show connections between them. Distance from the core signifies decreasing connectivity. Outliers, highlighted in color, are located in more central positions than expected by chance.

We next examined whether local adaptation outliers have higher connectivity than non-outliers, as might be expected given that outliers had a high probability of being eQTL. The putatively adaptive loci were more highly connected than the transcriptome-wide background (Fig 3B and Fig 4; *P* < 2 × 10^−16^, Wilcoxon rank-sum test). Although we found a negative non-linear trend between connectivity and *P*-values from the LFMM and PCAdapt analyses, there were no intermediate peaks that could be interpreted as intermediate levels of pleiotropy contributing most strongly to the observed difference between selection outliers and the transcriptome-wide background (S8 Fig). Connectivity also was higher for eQTLs than for non-eQTLs, as expected given that eQTLs were identified on the basis of their association with expression variation of other genes. Both outlier-sets also had higher connectivity after controlling for expression level and expression variance (linear model: *β*_LFMM_ = 0.16, *β*_PCAdapt_ = 0.16, *P* < 0.003), which can affect the probability of a gene being identified as highly connected [59]. Taken together, our results provide support for outlier genes being enriched for eQTLs and having greater connectivity than non-outlier genes. These results were also replicated using reads aligned to the *H*. *annuus* reference genome (S7 Fig), indicating that our findings are robust to potential errors arising from the de novo transcriptome assembly.

### Evolutionary simulations provide insight into the role of connectivity in local adaptation

Co-expression network-based connectivity has previously been associated with strong selective constraint, i.e. purifying selection, presumably reflecting the greater pleiotropy of the more highly connected genes [30–32]. We also found a signal of stronger purifying selection on more highly connected genes, as genetic diversity at both outlier and non-outlier genes was negatively correlated with connectivity (Table 1). However, we also found that outlier loci from local adaptation scans, i.e. genes bearing signatures of positive selection, had higher connectivity than non-outlier genes. Assuming connectivity is a proxy for pleiotropy [20,22,23], this result is consistent with the genes contributing most strongly to local adaptation being more pleiotropic than other genes.

**Table 1:** The relationship between expression features and genetic diversity. Shown are coefficients from multiple linear regression models and their 95% CIs. Last row shows the total variance explained by each model.

	Control genes		Selection outliers	
	θ_π_	θ_W_	θ_π_	θ_W_
Connectivity	–0.09 (–0.10, –0.07)	–0.12 (–0.13, –0.10)	–0.07 (–0.10, –0.04)	–0.09 (–0.13, –0.06)
Expression level	–0.08 (–0.09, –0.07)	–0.19 (–0.20, –0.18)	–0.15 (–0.18, –0.12)	–0.25 (–0.29, –0.22)
Expression variance	0.21 (0.20, 0.23)	0.22 (0.21, 0.23)	0.31 (0.28, 0.35)	0.30 (0.27, 0.33)
*R*^2^	0.06 (0.05, 0.07)	0.11 (0.10, 0.12)	0.13 (0.10, 0.15)	0.17 (0.15, 0.19)

**Fig 5:**
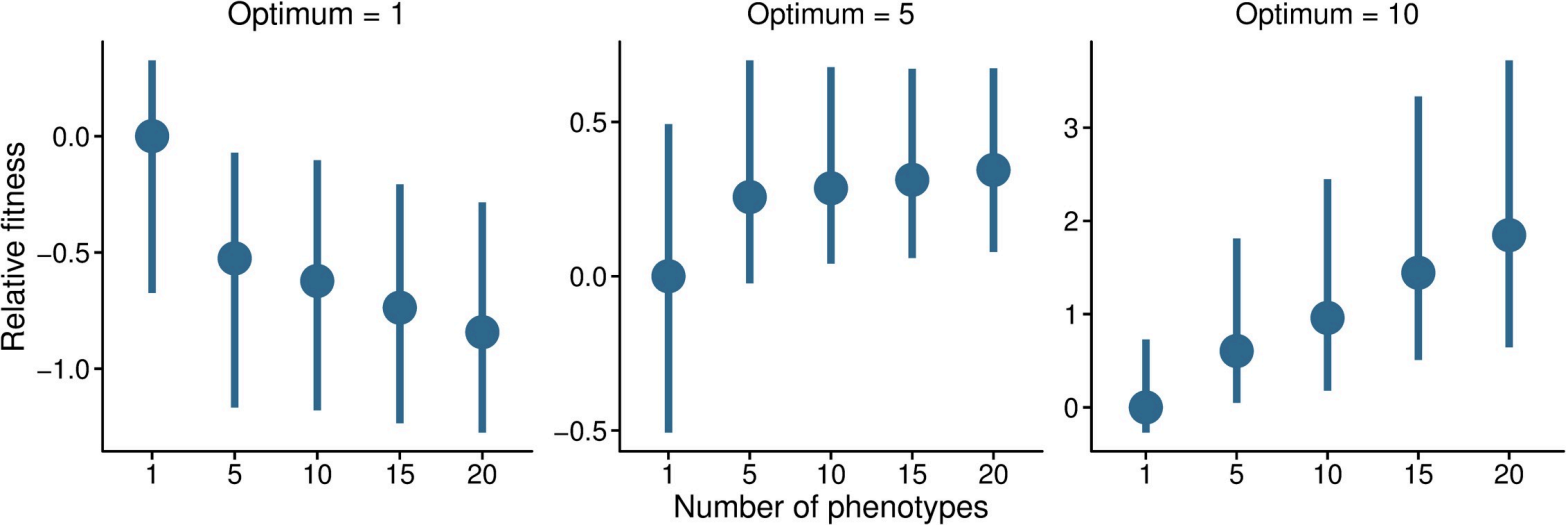
Simulated population fitness under different levels of pleiotropy, i.e. the number of phenotypes controlled by a single QTL (horizontal axis). Phenotypes start from an initial value 0 and selection acts to move them towards three different optima. Shown are medians and interquartile ranges (IQR) from 300 simulation. The fitness estimates were normalized in relation to the non-pleiotropic class (median = 0, IQR = 1).

The greater pleiotropy of local adaptation candidates may result from populations having experienced recent changes in selective environments, as *A*. *artimisiifolia* is likely to have experienced during range expansion. To assess this possibility, we conducted forward simulations to examine how population fitness is influenced by a single QTL controlling multiple phenotypes. Our simulations show that when phenotypes are close to a selective optimum (starting value 0, optimum 1), corresponding to populations that have not experienced recent shifts in the selective environment, loci with less pleiotropy will have a selective advantage (Fig 5). By contrast, when phenotypes move towards distant optima (5 and 10), pleiotropy becomes beneficial, with the benefit being greater the further the population is from the optimum (Fig 5). These results thus lend support to the hypothesis that highly pleiotropic genes are likely selected for when populations face new selective pressures.

## Discussion

The study of local adaptation has a long history in the field of evolutionary biology [60–65], which has resulted in the discovery of countless adaptive traits and loci over the years [66–73]. Yet fewer attempts have been made to understand constraints associated with local adaptation or the genetic architectures underlying adaptive traits [13,53,74–77]. Here, we identified genes that likely contribute to local adaptation by finding loci showing strong associations with environment variables (LFMM) and loci showing stronger than expected structure among populations (PCAdapt). These two methods use different approaches for handling the complications that population structure can have on identification of selection outliers. LFMM specifically searches for variants associated with environmental differences and strives to control for the confounding effects of population structure, whereas PCAdapt takes an environmentally naïve approach by searching for variants with particularly strong differentiation among populations. The outliers identified by both analyses bore several signatures of selection, including elevated differentiation between northern and southern populations, and skewed frequencies of polymorphic sites. Finding similar patterns from both analyses suggests that population structure is not a major factor underlying our findings, but rather different alleles have been selected for in different parts of the *A*. *artemisiifolia* range as a result of local adaptation.

Identifying loci that contribute to local adaptation does not, in itself, shed light on genetic constraints or the genetic architecture of local adaptation. Biparental QTL mapping has revealed several instances where loci controlling different phenotypes map to the same chromosomal location, suggesting that pleiotropic effects among genes responsible for local adaptation are common [10,12,14–16]. Such inferences are complicated, however, by the large confidence intervals of the QTLs, making it difficult to differentiate pleiotropy from linked loci [17]. For example, what first appeared to be a large-effect pleiotropic QTL in *Petunia*, turned out to be a number of small effect loci occupying the same chromosomal region [78].

In our analyses, we made use of the fact that transcriptome data, unlike genomic data, enable the use of genetic polymorphisms to search for evidence of adaptation, and to use expression polymorphisms to infer how genes affect the expression of other genes. Although the use of same samples to infer both genetic and expression variants could conceivably bias downstream analyses, we confirmed that our filtering effectively removed any systematic association between the two datasets. Moreover, the fact that measures of adaptation and connectivity were decoupled from variation in per-gene SNP densities, and the results from reads mapped to both *A*. *artemisiifolia* de novo transcriptome and *H*. *annuus* reference genome were highly similar, indicates that our results are not biased by mapping inaccuracies. We found that loci showing the clearest signatures of local adaptation also carried signatures of pleiotropy–these local adaptation candidates were far more likely than expected to be eQTL and were far more likely than expected to occupy central positions within the co-expression network. Although we lack functional validation for the identified local adaptation candidates, their enrichment among eQTLs and their central positions in co-expression network suggest that they have the potential to affect the expression of multiple other genes. As such, they are likely to be more pleiotropic than genes at the periphery of such networks [30–32]. This is similar to positions of genes in biochemical pathways, in which highly connected upstream genes are more pleiotropic, because mutations at those genes can affect all downstream phenotypes [20,23,25,26,28,79].

The theory arising from Fisher’s geometric model predicts that selection pressure experienced by large effect loci is dependent on how far phenotypes are from their selective optima. When population is well adapted to its environment, i.e. it is at the end of an “adaptive walk” [6], small effect loci are preferentially fixed by positive selection. In contrast, when a population is colonizing a new environment, large effect loci have a selective advantage [6]. To confirm that these predictions hold for pleiotropy, we used forward genetic models to examine the relationship between the number of phenotypes controlled by a locus and the distance from the phenotypic optimum. Our simulations, although limited in scope, revealed that the fitness benefits of pleiotropy can be positively associated with distance from a selective optimum. Importantly, the populations we studied are from regions into which *A*. *artemisiifolia* has recently expanded [37–39]. The expanding populations are likely to have experienced new selective environments and unlikely to have been at selective optima. Large-effect pleiotropic alleles may therefore have been more likely to contribute to adaptation than alleles of small or moderate effect. Recent theoretical work by Wang et al. [8] has suggested that alleles with intermediate levels of pleiotropy may have the highest adaptive potential due a balance between positive (increased effect size) and negative (cost of complexity) effects of pleiotropy. Frachon et al. [80] found empirical support for this model by showing that adaptation during an eight-year period was mediated by loci of intermediate pleiotropy in *Arabidopsis thaliana*. By contrast, we observed a nearly linear association between connectivity and *P*-values from the LFMM and PCAdapt analyses, suggesting that loci with highest levels of pleiotropy show more evidence of differential selection. Although we do not find direct support for the model by Wang et al., our results are not inconsistent with it. Rather, the difference between our findings and those of Frachon et al. may arise from the fact that the relative cost of pleiotropy should depend on the distance from the trait optima, thereby shifting the selective advantage towards higher levels of pleiotropy during a large-scale range expansion. We note, however, that the local adaptation candidates examined here were identified through scans of population genomic data, and thus are expected to be biased towards genes of larger phenotypic effects [81]. Therefore, our result of finding enrichment of pleiotropic effects among the local adaptation candidates might reflect both the recent change in the selective environment that *A*. *artemisiifolia* experienced during range expansion, as well as the potential for larger effect loci to be more pleiotropic.

Our finding of local adaptation candidates having high connectivity in co-expression networks does, at first glance, seem to conflict with the finding that more highly connected genes evolve under greater selective constraint, i.e. they bear stronger than expected signatures of purifying selection [30–32]. In fact, we also found a negative relationship between co-expression network connectivity and genetic diversity, consistent with stronger purifying selection on more highly connected genes [82]. However, these overall relationships between connectivity and purifying selection do not provide evidence that highly connected genes might not also be important for adaptation. The potential for more highly connected genes being responsible for recent adaptation is also seen in results from Des Marais et al. [83], who found that *A*. *thaliana* genes involved in cold responses were noticeably more connected in co-expression networks than drought response genes. In fact, Des Marais et al. hypothesize that cold stress is less frequent among their geographically wide sample of *A*. *thaliana* populations, mostly experienced by populations from high latitudes and altitudes, leading to sharper selection gradients than those imposed by drought stress. Our results suggest that the cold-response phenotypes studied by Des Marais et al. might have been distant from their trait optima due to recent colonization or climate change, thus giving a selective advantage to highly pleiotropic loci. In contrast, if the climate is more stable close to the species distribution core, drought-related phenotypes may be nearer their optimal values, leading to less pleiotropic alleles being selected for.

## Conclusions

By combining information from co-expression networks with genome scans for selection, we have gained novel insights into the role of pleiotropy in local adaptation. We showed that loci influenced by differential selection are highly connected with other loci, as they are located in central positions within the co-expression network. Our study species, *A*. *artemisiifolia*, has recently expanded its range in North America, which has likely led to more connected, and thus more pleiotropic, loci being favored by selection. We have also presented an approach for studying pleiotropy which has several attractive characteristics compared to genetic mapping. Most importantly, using connectivity as a proxy for pleiotropy has made our study independent of preselected phenotypes.

## Material and methods

*Ambrosia artemisiifolia* L. is self-incompatible [40], monoecious, and wind-pollinated [84] annual plant. It is native to North America, but it can be found as an invasive species on multiple continents [85,86]. As a pioneer species, *A*. *artemisiifolia* is found in high disturbance, low competition habitats [84]. *A*. *artemisiifolia* is now widely distributed across North America, and sedimentary pollen paleo-records, herbarium records, and genetic diversity estimates indicate that this was due to a recent westward range expansion that was facilitated by the expansion of agriculture [37–39].

### Sample collection and sequencing

We used seeds from 15 populations collected across a transect of 15 degrees of latitude and 7 degrees of longitude (Fig 1A, see Gorton et al. [42] for sampling details). From these populations, we selected 4–5 maternal families per population for sequencing. We stratified the seeds in moist silica sand and kept them in the dark at 4°C for 10 weeks to break seed dormancy. After stratification, we planted the seeds in 50-cell trays in BM2 germination mix (Berger), and let them germinate in a growth chamber under a 14-hour day and a day/night temperature of 22/20°C. We planted the seeds in a completely randomized design by including two to three seeds per cell. One week after planting, seedlings were randomly culled to one per cell. We grew the plants for 8 weeks, during which they were fertilized once, and watered as needed. After the 8 weeks, when each individual had between 8 and 12 leaves, we harvested the apical meristem and top four leaves from each plant. Tissue was collected on a single day over the course of one hour. We immediately flash froze the plant tissue in liquid nitrogen and then stored the samples at –80°C. Total RNA was extracted from ground tissue (~50 mg) using a RNeasy Plant Mini Kit (Qiagen). The 74 total RNA samples were submitted to University of Minnesota Genomics Center for library preparation and sequencing, where a stranded TruSeq RNA library was created for each sample. The libraries were pooled and sequenced over three lanes on Illumina HiSeq 2500 in high output mode with 125-bp paired-end reads (~220 million reads per lane).

### Transcriptome assembly

We constructed a de novo transcriptome for *A*. *artemisiifolia* to serve as a reference in the downstream analyses. After filtering out low quality reads and sequencing adapters with Trimmomatic [87], a total of 11.8 million read pairs from a single genotype (from Oklahoma) were used in the transcriptome assembly with Trinity [88]. After the primary transcriptome-assembly (116,642 contigs), we combined isoforms into contigs covering entire length of the genes. First, isoforms that were > 95% identical, potentially representing allelic splitting, were merged with CD-HIT [89], and the resulting contigs (93,857) used to make “SuperTranscripts” with Lace [90]. The final transcriptome used for alignments consisted of 58,644 contigs with a E90N50 (N50 estimated for contigs comprising 90% of the total expression) of 2510 bp. BUSCO v3 [43] was used to assess the completeness of our transcriptome assembly by searching for the presence of near-universal single-copy orthologs.

To evaluate whether information contained in a single genotype is sufficient to allow aligning reads from multiple populations, we assembled a second transcriptome using three genotypes from different parts of our sampling distribution (Minnesota, Missouri, and Louisiana). Although the three-genotype transcriptome included more contigs (102,126) than the one-genotype transcriptome, per-individual alignment percentages were ~4% lower with the larger assembly (89%– 97% for the one-genotype assembly vs. 86%– 91% for the three-genotype assembly). As the BUSCO analysis further indicated worse completeness for the three-genotype assembly (85% complete BUSCOs for the one-genotype assembly vs. 71% for the three-genotype assembly), we focused all further processing and analyses on the one-genotype assembly.

Besides constructing a de novo transcriptome for *A*. *artemisiifolia*, we utilized a reference genome of a closely related species from the same family (Asteraceae), *Helianthus annuus* (common sunflower) [45]. Our assumption was that sequence divergence at genic regions is low enough that a majority of *A*. *artemisiifolia* reads can be successfully aligned to the *H*. *annuus* genome. This approach allowed us to find putatively novel transcripts in *A*. *artemisiifolia*, as well as to assess whether complications related to build-quality of the transcriptome or alignment of reads to a distant reference (e.g. higher reference allele bias [91]) results in unreliable inferences. For this reason, we did not use the *H*. *annuus* refence to construct a genome-guided assembly, as this can bias the resulting transcriptome towards the reference genome [92].

### Sequence processing and alignments

Low quality reads and sequencing adapters were removed with Trimmomatic [87], and the surviving reads first aligned to our transcriptome assembly with STAR [93]. We used STAR because it allows for gapped alignments across the SuperTranscripts [90]. Picard tools (https://broadinstitute.github.io/picard/, last accessed October 14, 2019) was used to add read group information and remove duplicated reads. To focus our analyses on high quality regions of the transcriptome, we removed reads aligning to contigs having median read-count < 1 across individuals. This additional filtering step left 38,111 contigs, each between 201 and 25,360 bp in length (median of 883 bp). As each SuperTranscript potentially represents an expressed gene [90], we refer to these contigs as genes throughout this paper. For expression analyses, per-gene read-counts produced by STAR were normalized with variance stabilizing transformation in DESeq2 [94].

The quality-filtered reads were next aligned to the *H*. *annuus* reference genome with STAR, using slightly relaxed alignment criteria (30% of the read-pair, i.e. 75 bp, needed to align with maximum of 5 mismatches). This allowed us to retain a useful number of reads (between 57% and 70% per individual), while ensuring unique alignments.

### Variant calling

Variant calling for both datasets was done with FreeBayes [95], using only reads with mapping quality over 30. VCFtools [96] was used to filter the resulting VCF-file. We only kept biallelic SNPs with the following requirements: site quality ≥ 30, genotype quality ≥ 20, read coverage ≥ 6, < 80% heterozygosity across individuals, and missing data in < 20% of individuals. For LFMM, PCAdapt, and eQTL analyses, minor allele frequency ≥ 0.05 was also required. As our study is conducted on transcriptome data, there is a direct correlation between expression level and sequencing coverage. To confirm that our filtering sufficiently accounts for the influence of expression level on SNP-calling, we tested for an association between sequencing coverage and the called genotype. The sequencing coverage can especially affect the calling of heterozygous sites [97], so besides coding the genotypes based on their number of non-reference alleles (0, 1, 2), we treated the genotype as a binary trait (homozygous = 0, heterozygous = 1). With both approaches, the genotype and coverage were effectively uncorrelated in the filtered data ($R_{0,1,2}^{2}$ = 0.0002, $R_{0,1}^{2}=8.5\times 10^{-5}$), indicating that our transcriptome data can be used to find both genetic and expression variants without an inherent bias.

### Genetic diversity and population structure

We examined genetic diversity within and between populations by estimating four summary statistics with ANGSD [98]: nucleotide diversity θ_π_ [99], Watterson estimator θ_W_ [100], Tajima’s *D* [46], and *F*_ST_ [101]. PCAngsd [47] was used to assess genetic relatedness among the study individuals by conducting a principal component analysis (PCA) and an admixture analysis.

### Selection scan

To find genes potentially underlying adaptive phenotypes, we first searched for associations between SNP allele frequencies and environmental variables with latent factor mixed models in an R package LFMM 2 [48]. We characterized environments of the populations by extracting all 19 BIOCLIM variables for each sampled location at a spatial resolution of 5 minutes [52]. Due to high collinearity between the BIOCLIM variables, we conducted a PCA to capture the main axes of variation in the environment data. The first four PCs, which explained more than 97% of the variance in climate across the sampled locations (S3 Table), were used as predictors in the LFMM models. Based on cross validation error among different ridge penalty models, four latent factors (*K* = 4) were chosen to account for population structure in the genotype data. As a second approach to find genes potentially contributing to local adaptation, we used an allele frequency differentiation based method implemented in an R package PCAdapt [49]. PCAdapt utilizes PCA to identify population structure in the dataset and searches for higher than expected associations between SNPs and the PC axes. As a PCA-based scree plot (estimated from LD-pruned data: *R*^2^ < 0.1 within 200 SNPs) indicated that there were around four main components in the SNP-data (S9 Fig), we used *K* = 4 with PCAdapt. We identified SNPs with higher than expected associations with the environment (LFMM) and genotype (PCAdapt) PCs by transforming *P*-values to false discovery rate based *q*-values [102]. SNPs with *q*-value lower than 0.05 were considered putatively adaptive.

### Analysis of outlier loci

Genes housing the outlier SNPs from either LFMM or PCAdapt analyses were examined in more detail to gain insight into selection acting on these loci. As both LFMM and PCAdapt strive to find footprints of spatially varying selection, and because adaptive phenotypes in *A*. *artemisiifolia* show strong latitudinal clines in the native range [42,103], we conducted this analysis on populations from opposite edges of our sampling distribution, Minnesota and Louisiana. First, we quantified population differentiation using a relative measure *F*_ST_ [101,104] and an absolute measure *d*_XY_ [105,106]. We then estimated a neutrality measure, Fay and Wu’s *H* [55], for the genes. By comparing θ_π_ to a diversity measure based on derived variants, *H* can be used to find regions with an excess of high-frequency derived alleles–a signal of recent positive selection. The likely ancestral state of alleles was defined by using *H*. *annuus* as an outgroup. The *A*. *artemisiifolia* transcriptome was aligned against the *H*. *annuus* reference genome with MUMmer4 [107], and variants in regions showing one-to-one alignments used to estimate a normalized version of the *H* statistic [108]. To further assess whether the outliers have experienced selective sweeps, we estimated composite likelihood ratios (CLR) [56,57] for each gene. CLR utilizes a likelihood ratio framework to explicitly test for skews in the site frequency spectrum due to hitchhiking. The CLR calculations were done with SweepFinder2 [109], using a custom grid search that encompassed each variable site within a gene. The transcriptome-wide site frequency spectrum was used as the neutral allele frequency distribution.

### eQTL mapping and co-expression network

To gain insight into gene regulatory networks in *A*. *artemisiifolia*, we used two approaches for quantifying the connectivity of genes: one based on expression QTL mapping (eQTL) and one based on co-expression networks. We used a PCA to test for the presence of population structure in the expression data. A scree plot indicated that there were around two main components in the data (S10 Fig), of which PC1 appears to be related to a north-south gradient among populations (S11 Fig). We therefore conducted a regression-based correction by fitting the following linear-model to the data: expression level = PC1 + PC2, and then used the resulting residuals in the downstream-analyses. This correction was done for both de novo transcriptome and reference-genome aligned data (S11 Fig and S12 Fig).

eQTLs were mapped by searching for associations between SNP-genotypes and expression levels with an R package Matrix eQTL [110]. To account for population structure in the genotype-data, admixture-proportions (*K* = 4) were used as covariates in the eQTL models. As our eQTL mapping is done with transcriptome data, and thus all SNPs are presumably in coding areas of the genes, we defined eQTLs as genes carrying SNPs with significant (*q* < 0.05) genotype-expression associations, which likely represent *trans*-regulatory variants. This is in contrast to many other studies [e.g. 30,111], where eQTLs are mainly thought of as *cis*-regulatory variants residing in intergenic regions. Additionally, eGenes were defined as genes whose expression was influenced by the eQTLs.

We then constructed a co-expression network by grouping genes with similar expression patterns into modules. In systems biology, co-expression networks are commonly constructed by comparing samples collected across different tissue types or treatments from a single genotype [112]. Here, however, we utilized expression differences between genetically distinct individuals, as these patterns are likely better at reflecting regulatory variation existing in natural populations [30,31,58], and thus better able to capture variation associated with phenotypic variation than networks constructed from samples of a single genotype [113]. The co-expression network was constructed with an R package WGCNA [59]. We used a soft thresholding power of six to calculate adjacencies for a signed network. Topological overlap matrix (TOM) and dynamic-tree cut algorithm were used to detected network modules. The connectivity for each gene was estimated as the sum of adjacencies between the focal gene and other genes in the network.

### Forward simulations

We used forward genetic models in SLiM 3 [114] to explore adaptive constraints associated with connectivity. Assuming that connectivity is a proxy for pleiotropy [20,22,23,30–32], we simulated a single additive QTL region controlling varying number of phenotypes. The simulated region was 50 kb long, with a 10 kb “genic region” in the middle. Mutation rate was 1.5 × 10^−8^ per site per generation and recombination rate 5 × 10^−8^ per site per generation. We established a single random-mating population of *N =* 10,000 diploid individuals. The simulation was run 10*N* generations to approach equilibrium, after which 10% of new mutations in the genic region and 1% elsewhere had a fitness effect. Individual fitness *w* was estimated using a multivariate Gaussian fitness function:

$$w=exp\left(-\frac{1}{2}\sum_{i=1}^{n}\frac{(z_{i}-z_{o}{)}^{2}}{2Vs}\right)$$

where *z_i_* is the observed phenotypic value for a trait *i*, *z_o_* is the optimum phenotypic value, and *Vs* is the variance of the fitness profile. Here, we assumed a *Vs* = 10, corresponding to a moderate stabilizing selection around the selective optimum [115]. Mutational effects were drawn from a multivariate Gaussian distribution with a mean of 0, standard deviation of 1, and covariance of 0.1. The choice of standard deviation allows for a wide range of mutational effects, and the covariance parameter results in weak correlation among phenotype. This correlation is meant to simulate trait-modularity observed in natural organisms, wherein a mutation is more likely to influence a set of related traits to the same direction than a set of unrelated traits to random directions [3,8,18]. Although the correlation between phenotypes increases synergistic pleiotropy, our results stay qualitatively the same without the covariance parameter (S13 Fig). We examined models differing in their level of pleiotropy and phenotypic optima. The QTL controlled *n* = 1, 5, 10, 15, or 20 phenotypes. The phenotypes had an initial value of 0 and we defined the optimum as *z_o_* = 1, 5, or 10. To assess the effects of recent adaptation to a new optimum, the mean fitness was recorded 0.1*N* generations after the start of selection. Models with each parameter combination were repeated 300 times.

## Supporting information

S1 Fig Temperature and precipitation variation across our *A*. *artemisiifolia* sampling distribution. Environment data from WorldClim (https://www.worldclim.org), map data from GADM (https://gadm.org).
(PDF)

S2 Fig First two genotype-PCs estimated from reads aligned to the *H*. *annuus* reference genome. The proportion of variance explained by the PCs is shown in brackets.
(PDF)

S3 Fig Admixture proportions estimated from reads aligned to the *H*. *annuus* reference genome. (PDF)

S4 Fig Genetic differentiation between populations from Minnesota and Louisiana. *F*_ST_ and *d*_XY_ distributions of the selection outliers, eQTLs and eGenes are compared against the rest of the transcriptome. The horizontal lines mark the medians of the control genes. Reads were aligned to the *A*. *artemisiifolia* de novo transcriptome.
(PDF)

S5 Fig Genetic diversity at candidate genes. θ_π_ and θ_W_ distributions of the selection outliers, eQTLs and eGenes are compared against the rest of the transcriptome for two datasets. The horizontal lines mark the medians of the control genes. Reads were aligned to the *A*. *artemisiifolia* de novo transcriptome.
(PDF)

S6 Fig The distributions of Fay and Wu’s *H* and CLR estimates compared between the two outlier-sets. Horizontal lines mark the medians of the datasets. Reads were aligned to the *A*. *artemisiifolia* de novo transcriptome.
(PDF)

S7 Fig Gene network topology at the candidate genes using reads aligned to the *H*. *annuus* reference genome. **A**: The percentage of eQTLs and eGenes found among the LFMM and PCAdapt outliers compared against all genes. Error bars show 95% bootstrap-based CIs. **B**: Connectivity measures at candidate genes. The horizontal line marks the median of the control genes.
(PDF)

S8 Fig Association between connectivity and *P*-values from LFMM and PCAdapt analyses. Shown are model fit from generalized additive models (GAM).
(PDF)

S9 Fig A scree plot from SNP-based PCA. Reads were aligned to the *A*. *artemisiifolia* de novo transcriptome.
(PDF)

S10 Fig A scree plot from expression-based PCA. Reads were aligned to the *A*. *artemisiifolia* de novo transcriptome.
(PDF)

S11 Fig First two principal components from uncorrected and corrected expression-level PCA. Reads were aligned to the *A*. *artemisiifolia* de novo transcriptome.
(PDF)

S12 Fig First two principal components from uncorrected and corrected expression-level PCA. Reads were aligned to the *H*. *annuus* reference genome.
(PDF)

S13 Fig Simulated population fitness with no covariance between phenotypes. Phenotypes start from an initial value 0 and selection acts to move them towards three different optima. Shown are medians and interquartile ranges (IQR) from 300 simulations. The fitness estimates were normalized in relation to the non-pleiotropic class (median = 0, IQR = 1).
(PDF)

S1 Table Pairwise *F*_ST_ estimates among the sampled populations. (PDF)

S2 Table Genetic diversity estimates for the study populations. (PDF)

S3 Table PCA loadings for the 19 BIOCLIM variables and the proportion of variance explained (PVE) by the PCs. (PDF)
