# Supplementary figures and images for: Pleiotropy facilitates local adaptation to distant optima in common ragweed (Ambrosia artemisiifolia)
Source: PLoS Genet. 2020 Mar 25;16(3):e1008707. doi: 10.1371/journal.pgen.1008707 (PMC7135370; doi:10.1371/journal.pgen.1008707)

Annual mean temperature (°C)

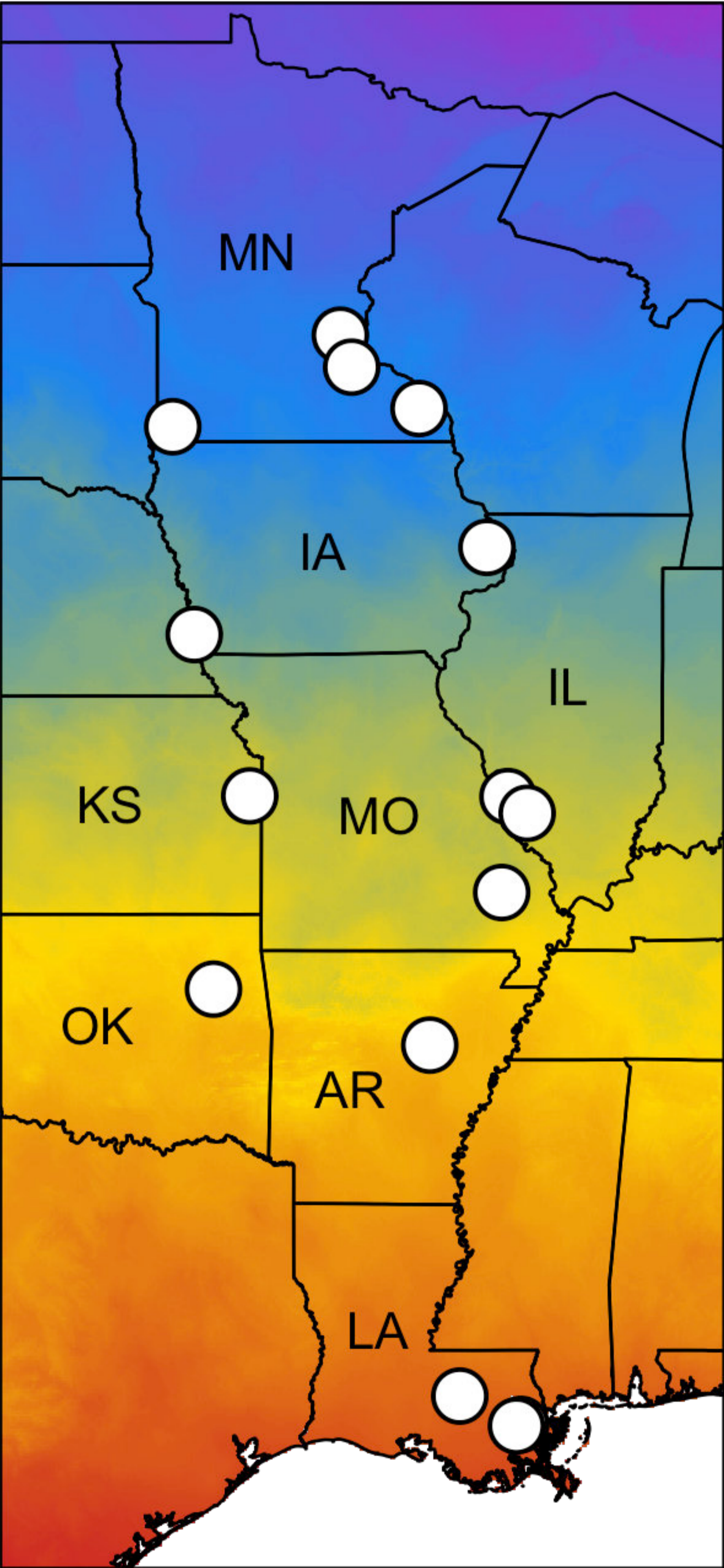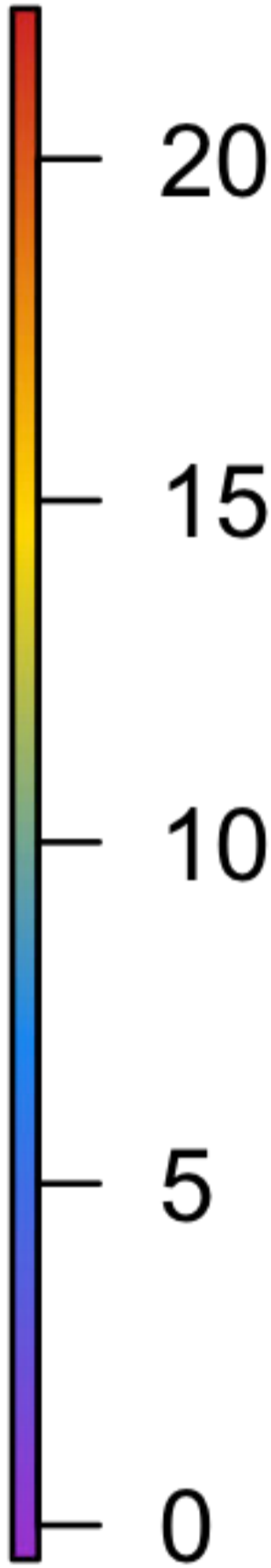

Annual precipitation (cm)

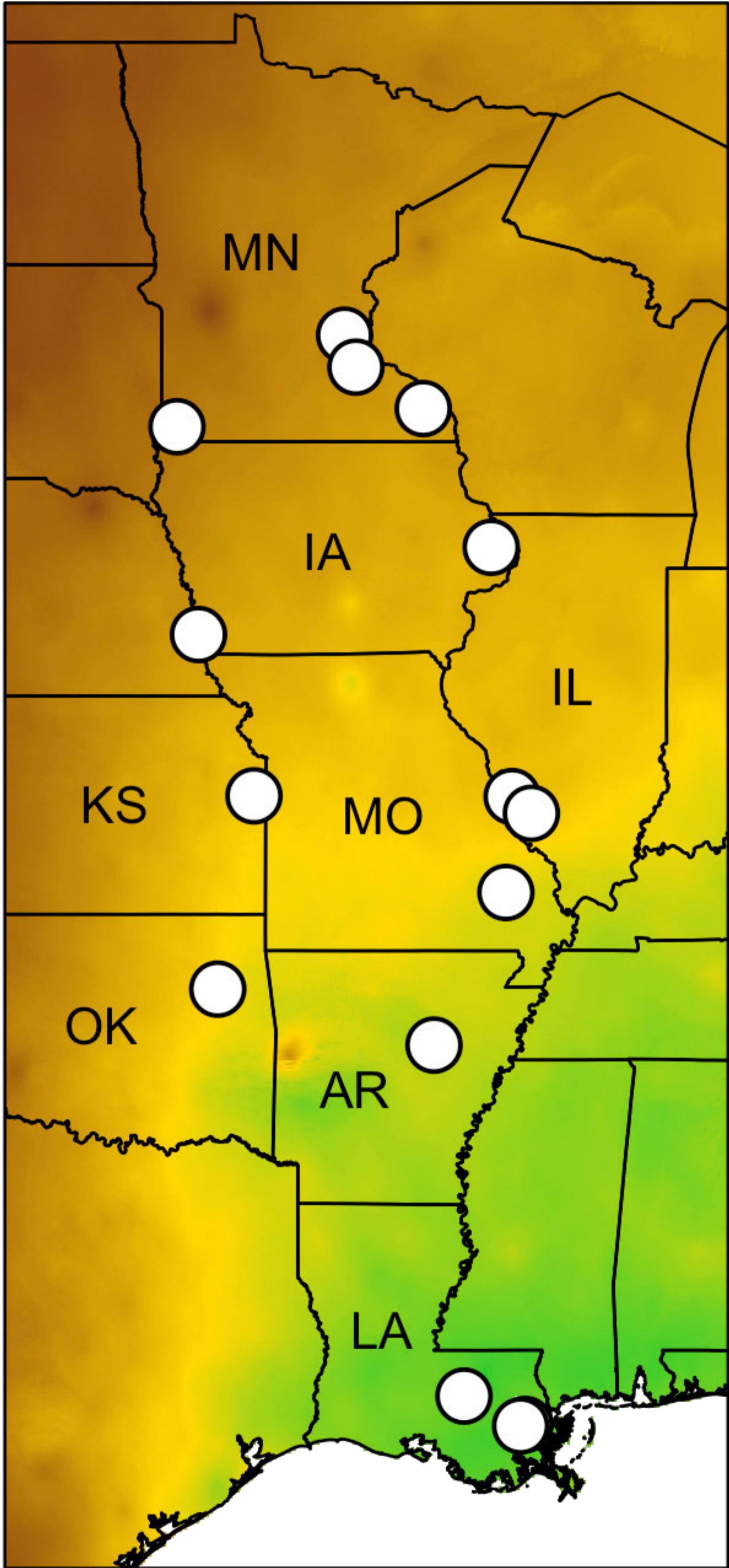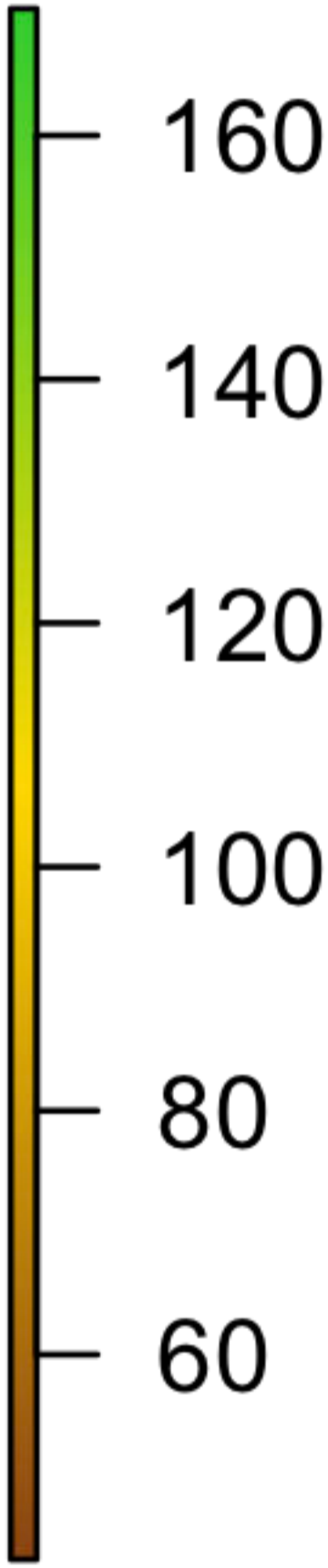

Supplement: S1 Fig — Environment data from WorldClim (https://www.worldclim.org), map data from GADM (https://gadm.org). (PDF) [file pgen.1008707.s001.pdf]

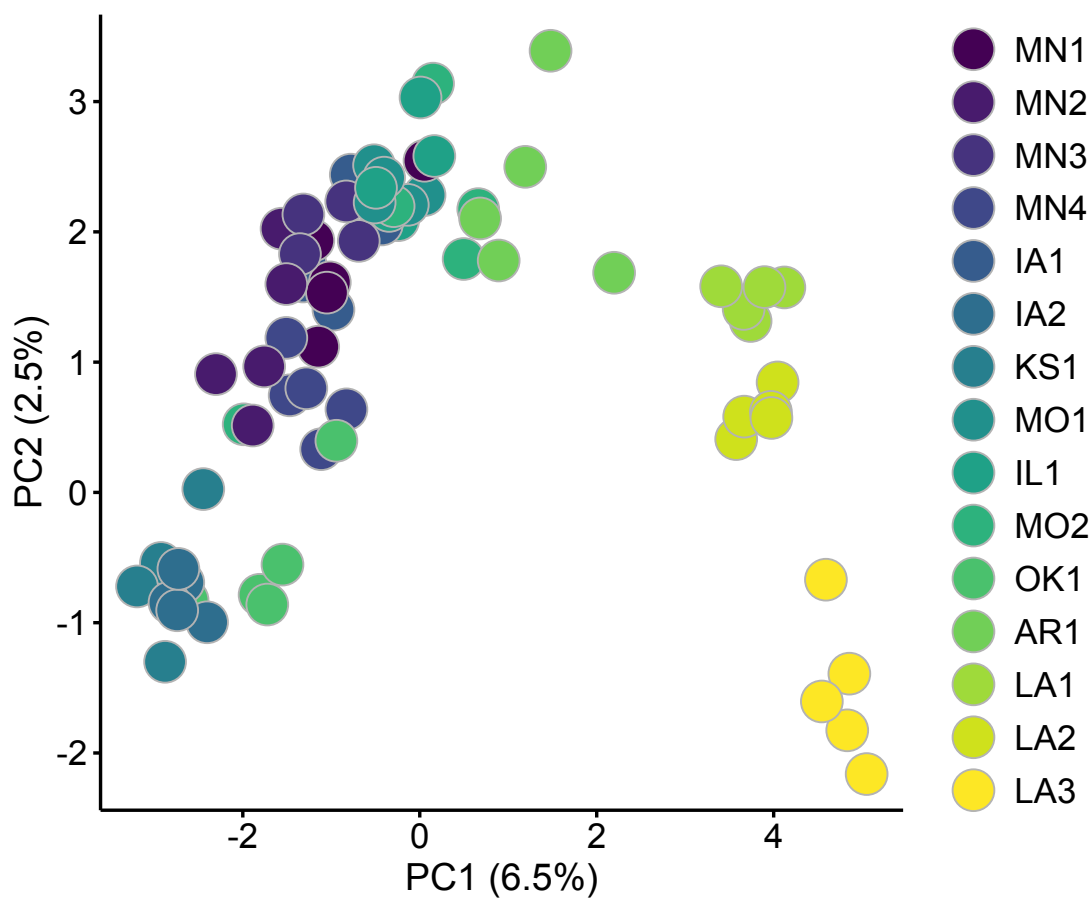

Supplement: S2 Fig — The proportion of variance explained by the PCs is shown in brackets. (PDF) [file pgen.1008707.s002.pdf]

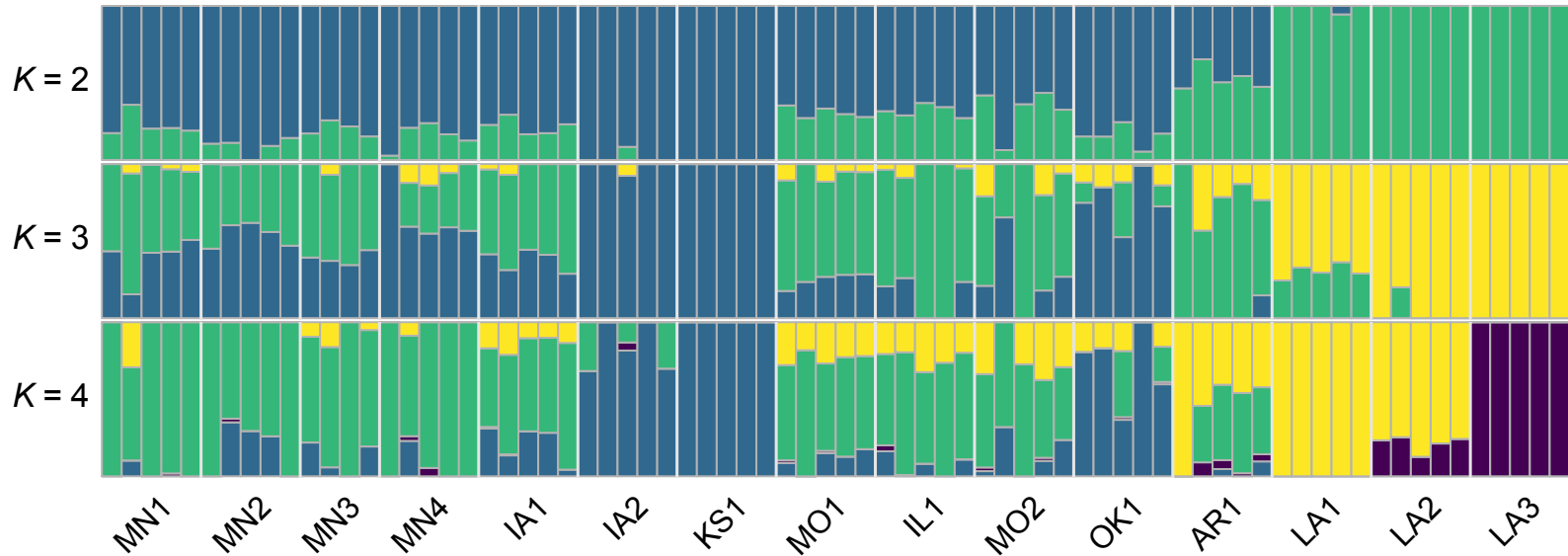

Supplement: S3 Fig — (PDF) [file pgen.1008707.s003.pdf]

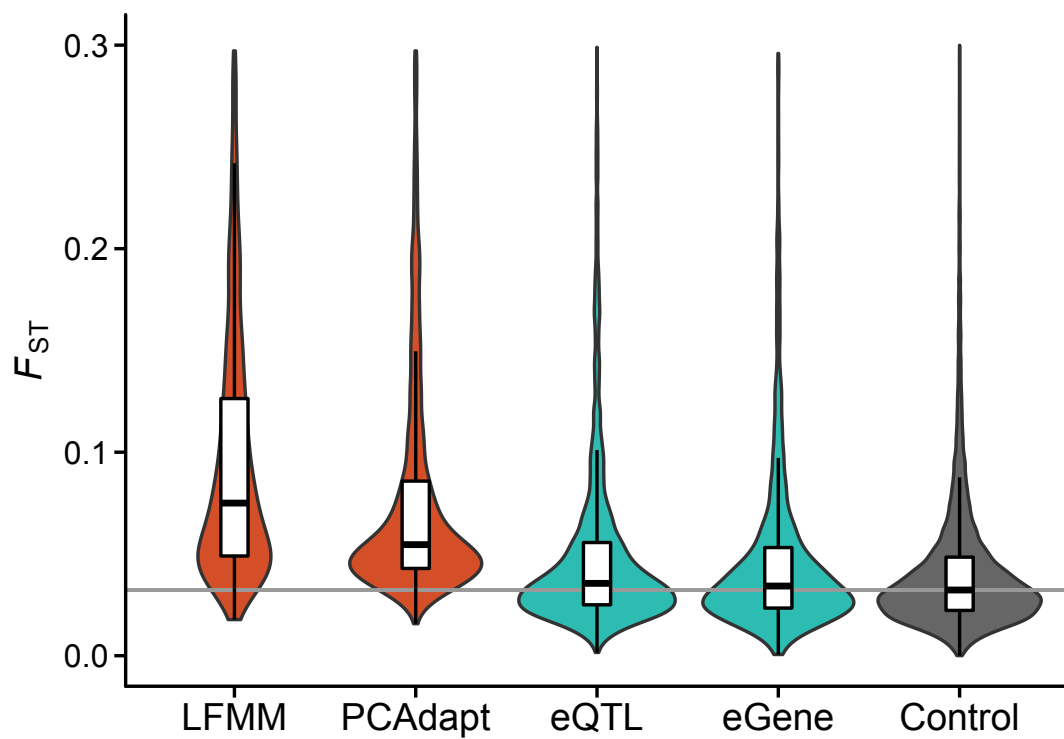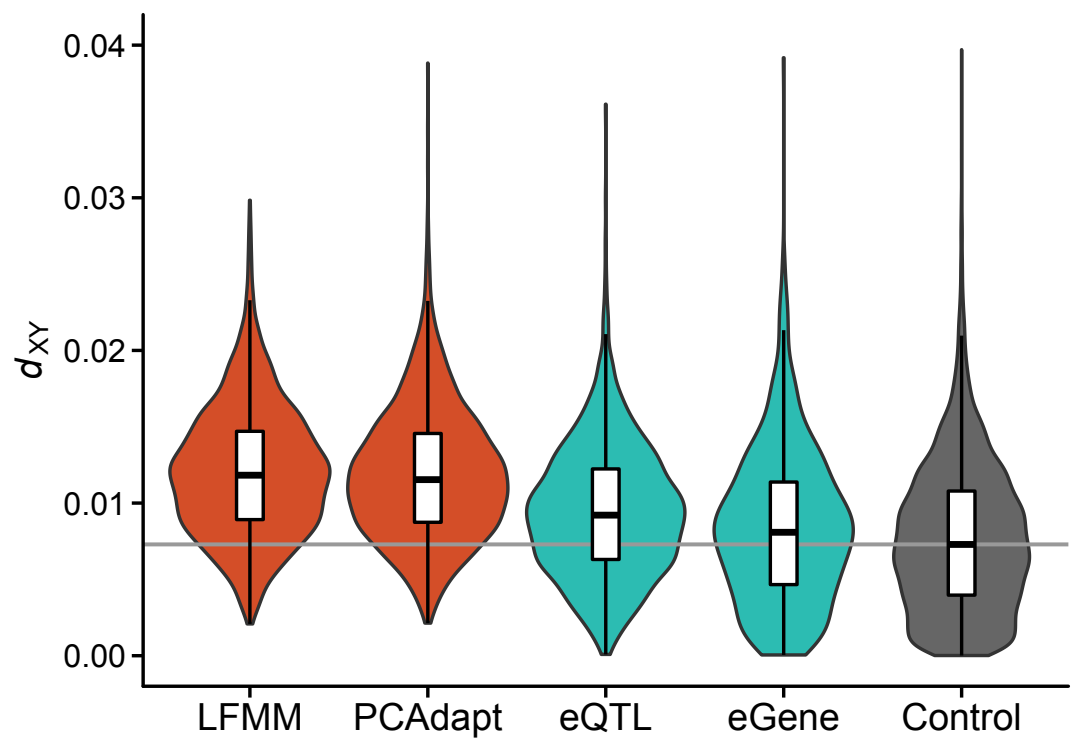

Supplement: S4 Fig — FST and dXY distributions of the selection outliers, eQTLs and eGenes are compared against the rest of the transcriptome. The horizontal lines mark the medians of the control genes. Reads were aligned to the A. artemisiifolia de novo transcriptome. (PDF) [file pgen.1008707.s004.pdf]

Minnesota

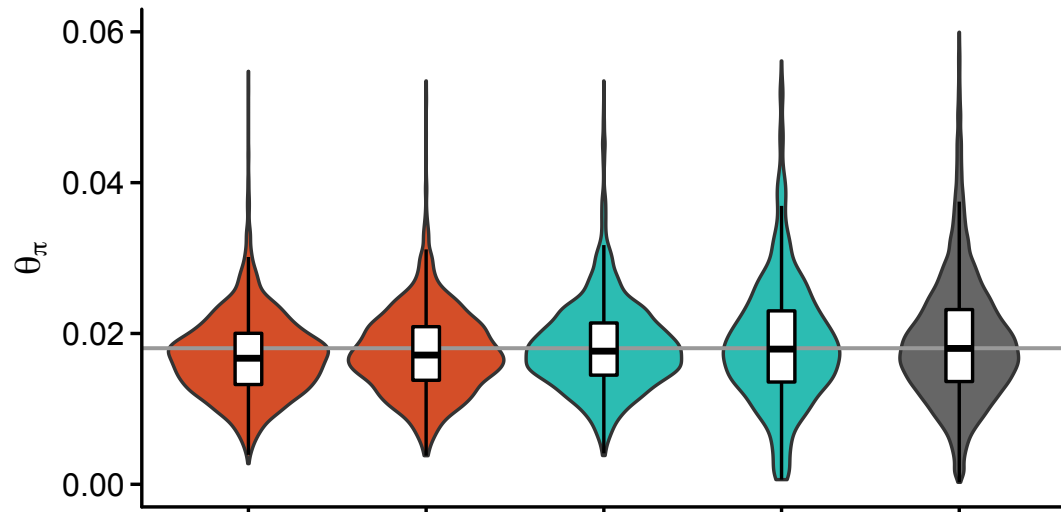

Louisiana

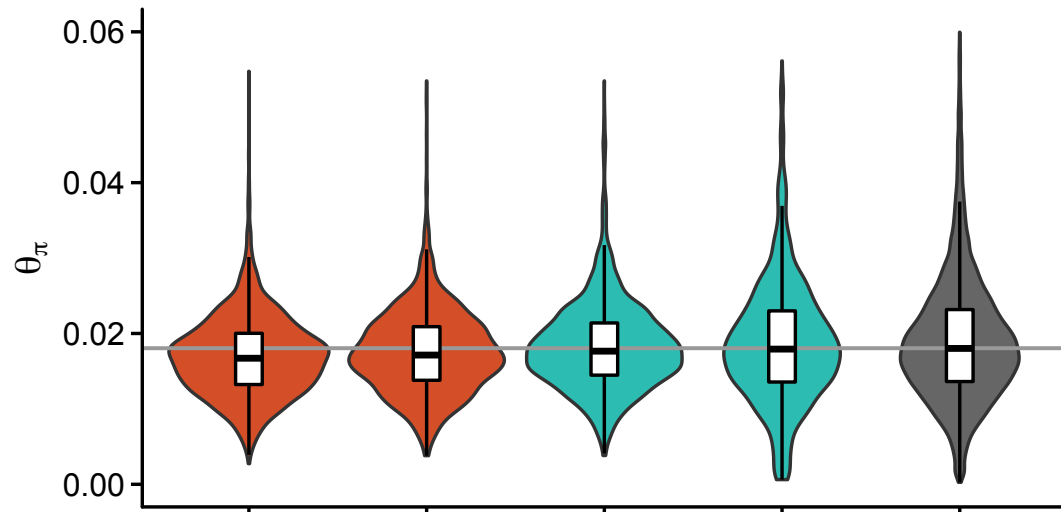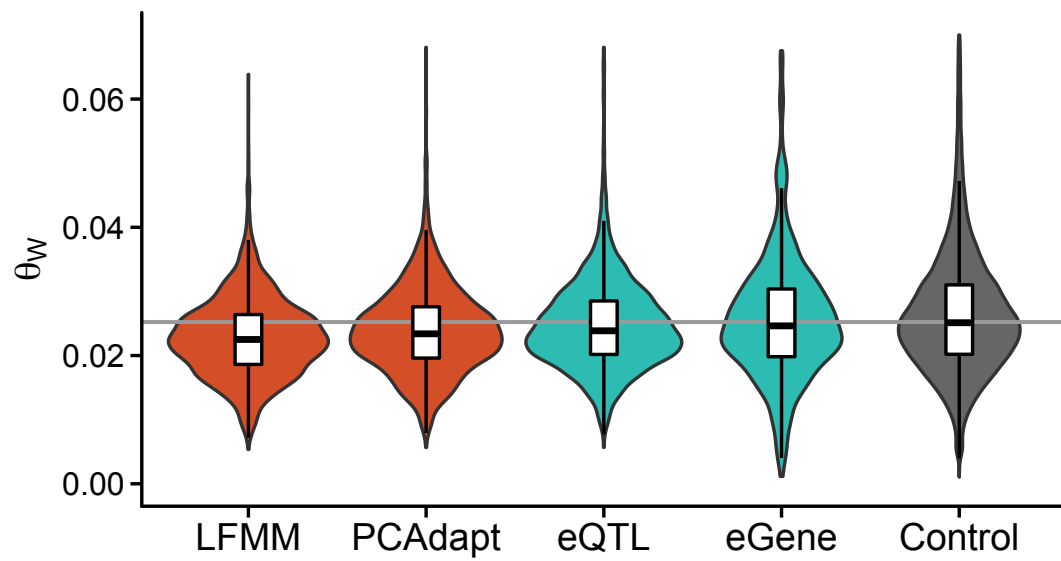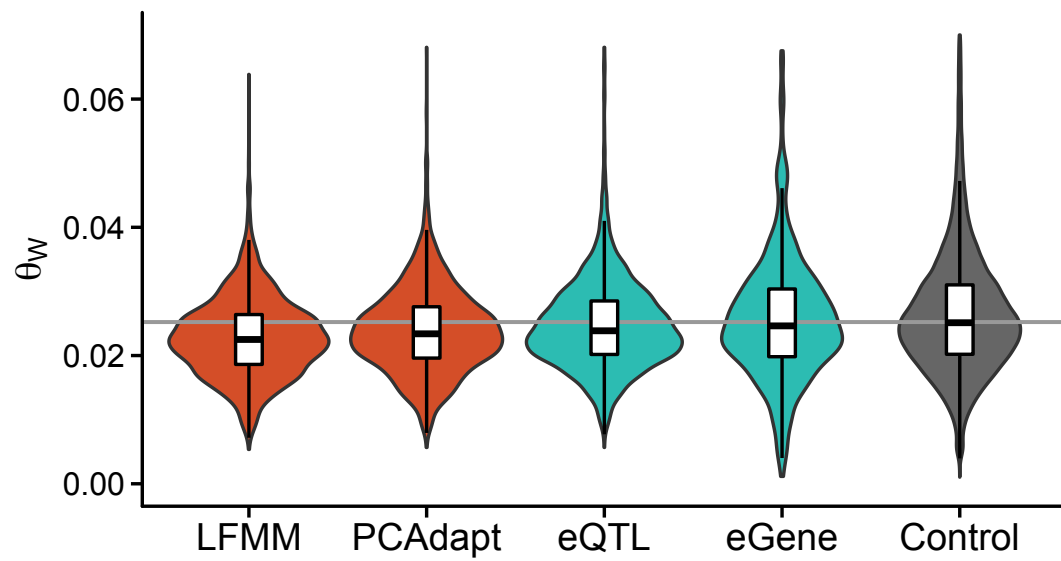

Supplement: S5 Fig — θπ and θW distributions of the selection outliers, eQTLs and eGenes are compared against the rest of the transcriptome for two datasets. The horizontal lines mark the medians of the control genes. Reads were aligned to the A. artemisiifolia de novo transcriptome. (PDF) [file pgen.1008707.s005.pdf]

Minnesota

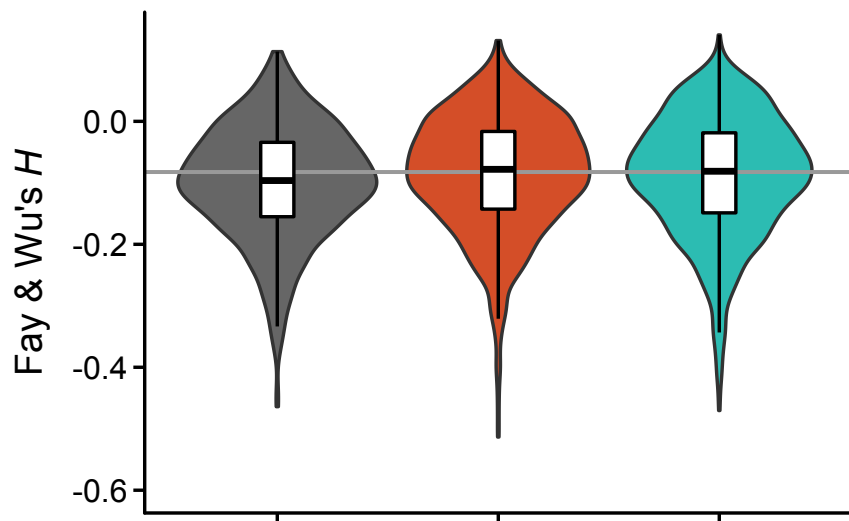

Louisiana

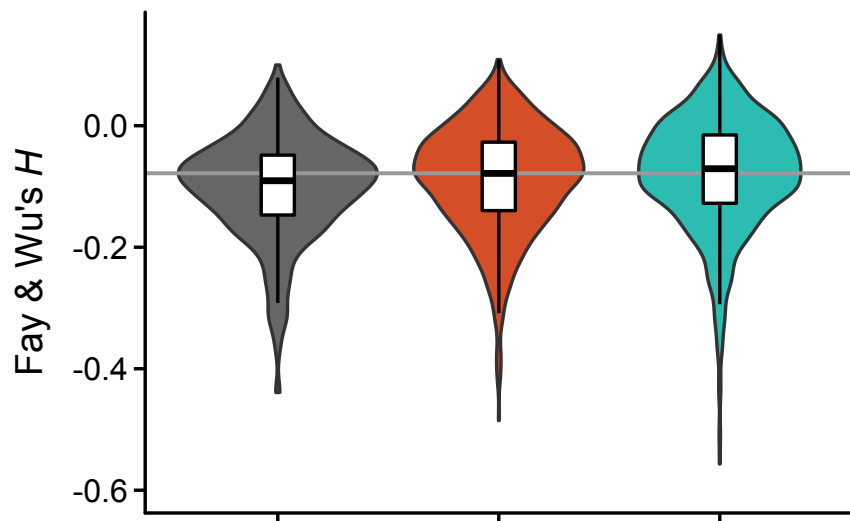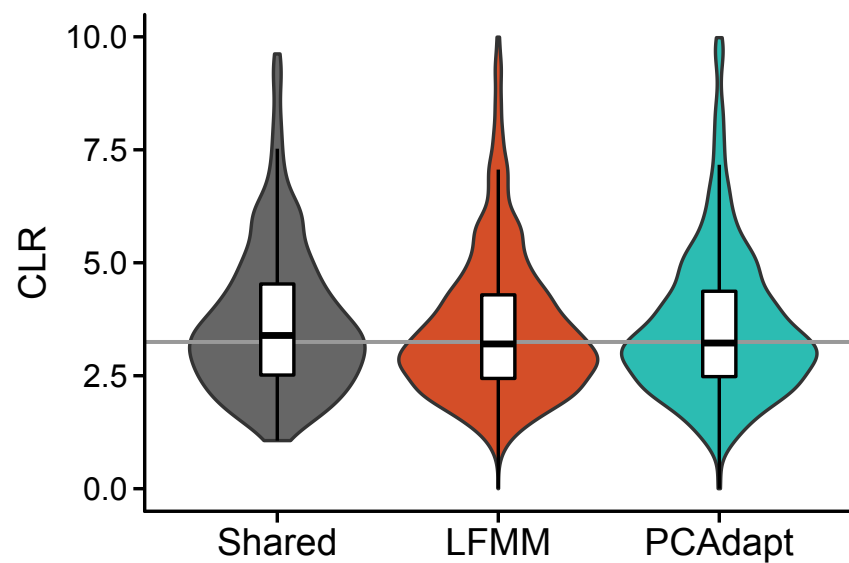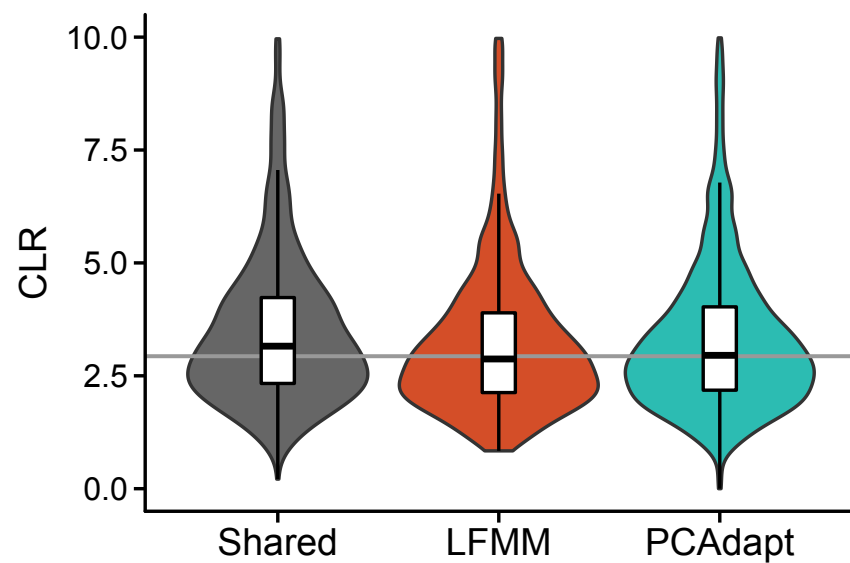

Supplement: S6 Fig — Horizontal lines mark the medians of the datasets. Reads were aligned to the A. artemisiifolia de novo transcriptome. (PDF) [file pgen.1008707.s006.pdf]

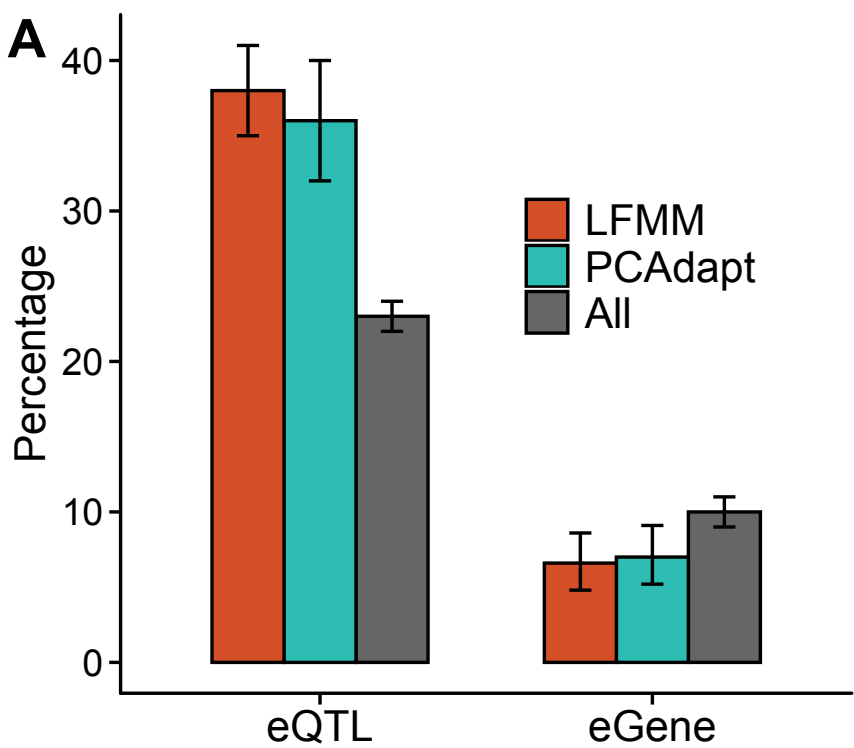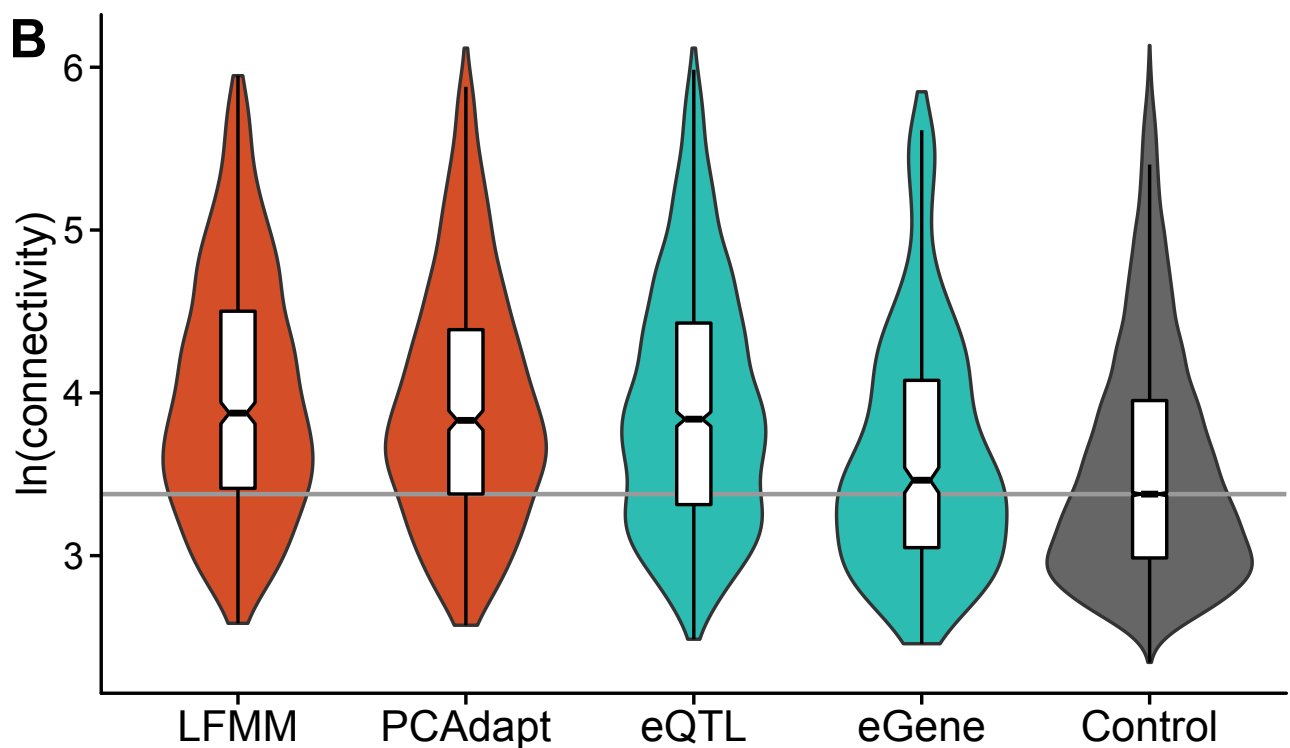

Supplement: S7 Fig — A: The percentage of eQTLs and eGenes found among the LFMM and PCAdapt outliers compared against all genes. Error bars show 95% bootstrap-based CIs. B: Connectivity measures at candidate genes. The horizontal line marks the median of the control genes. (PDF) [file pgen.1008707.s007.pdf]

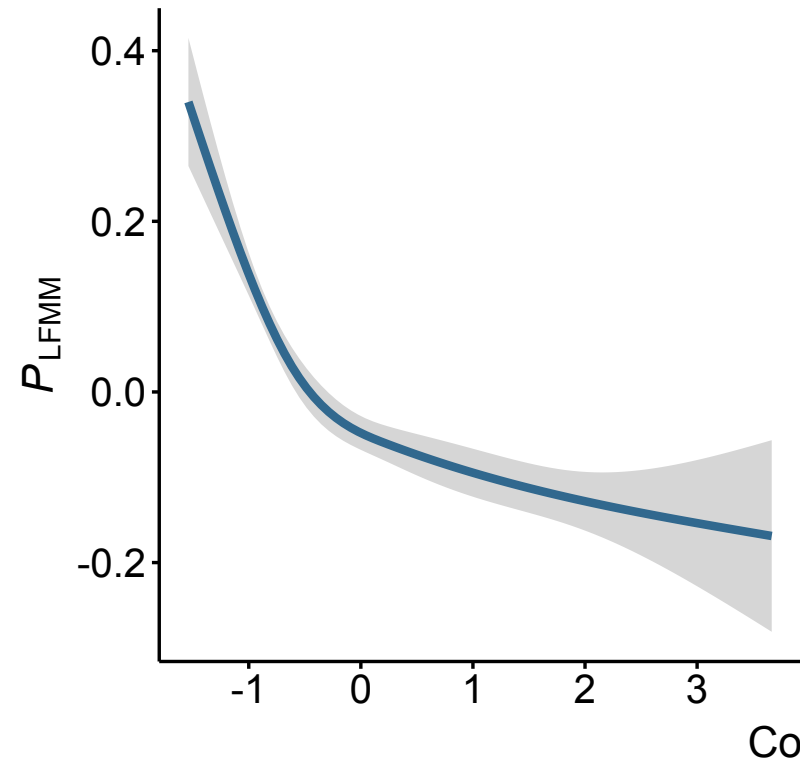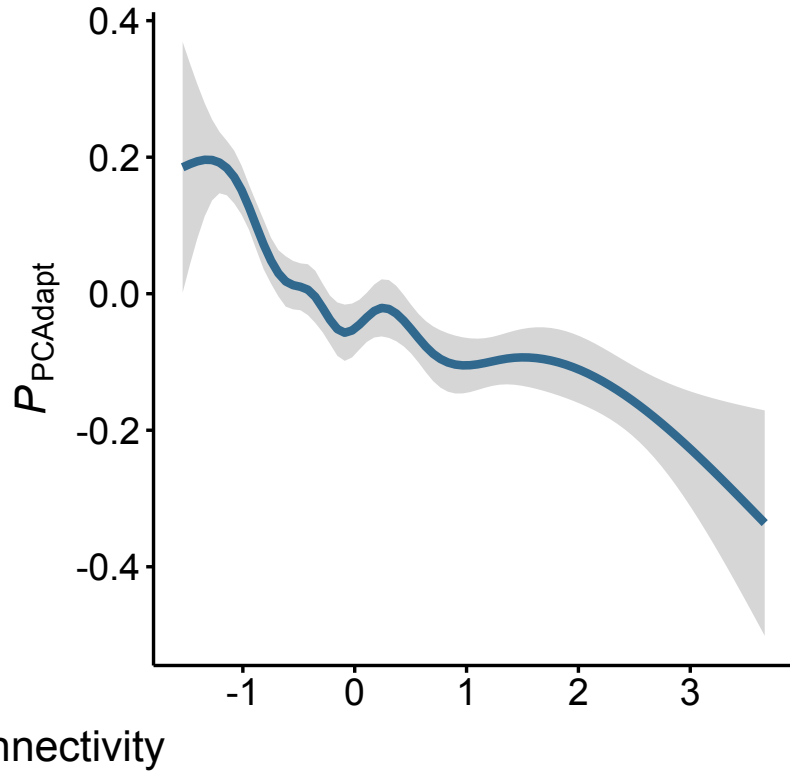

Supplement: S8 Fig — Shown are model fit from generalized additive models (GAM). (PDF) [file pgen.1008707.s008.pdf]

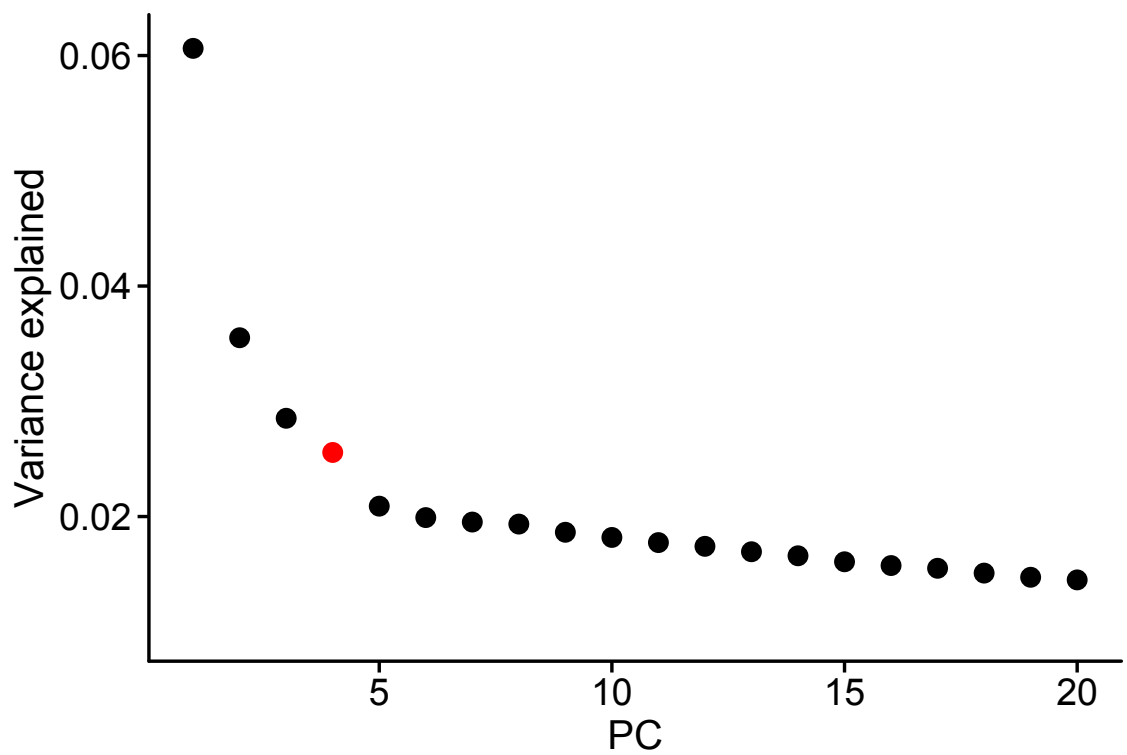

Supplement: S9 Fig — Reads were aligned to the A. artemisiifolia de novo transcriptome. (PDF) [file pgen.1008707.s009.pdf]

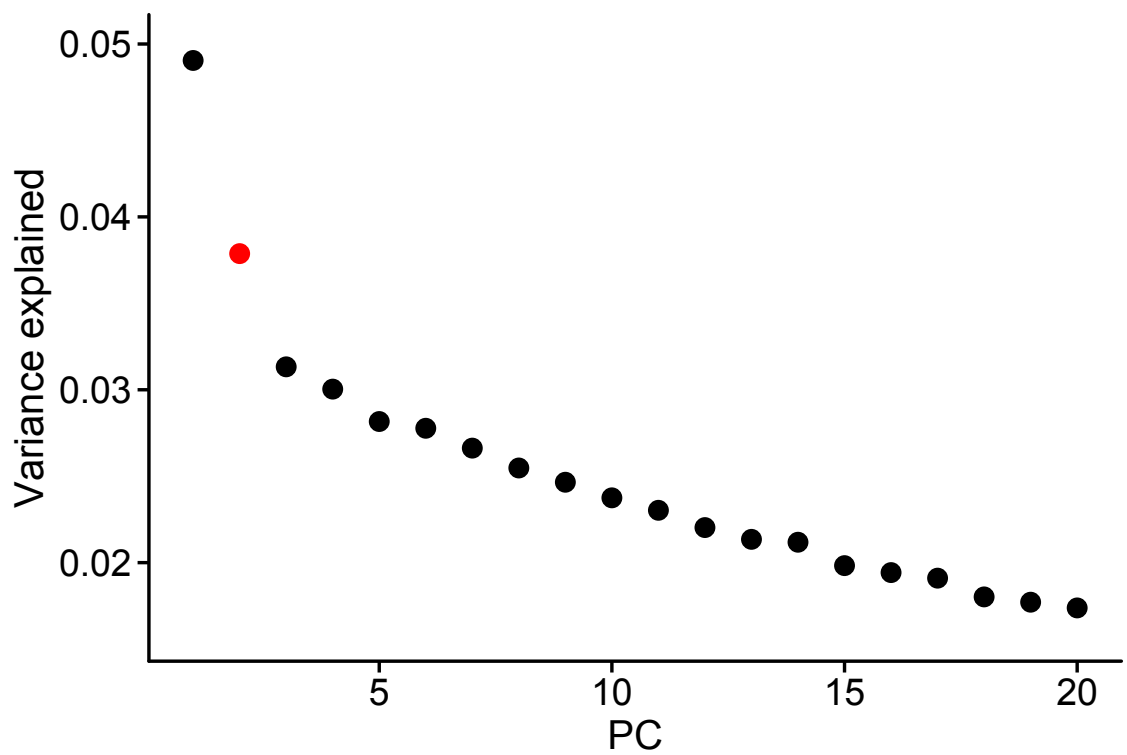

Supplement: S10 Fig — Reads were aligned to the A. artemisiifolia de novo transcriptome. (PDF) [file pgen.1008707.s010.pdf]

Uncorrected

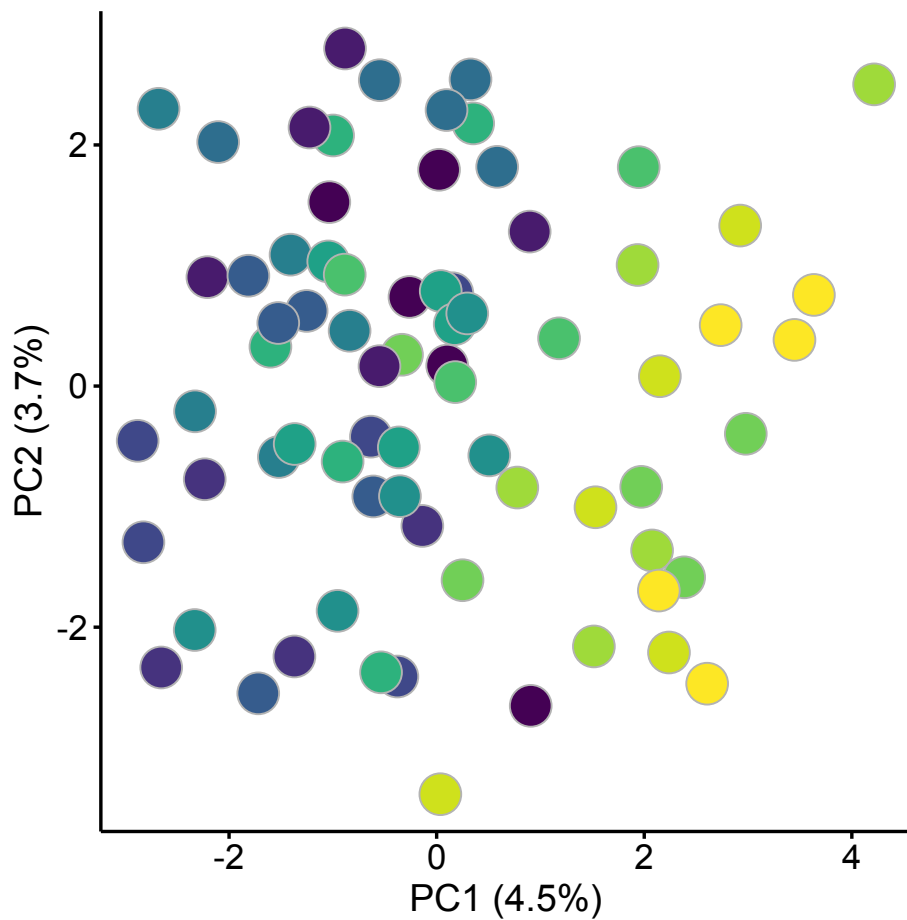

Corrected

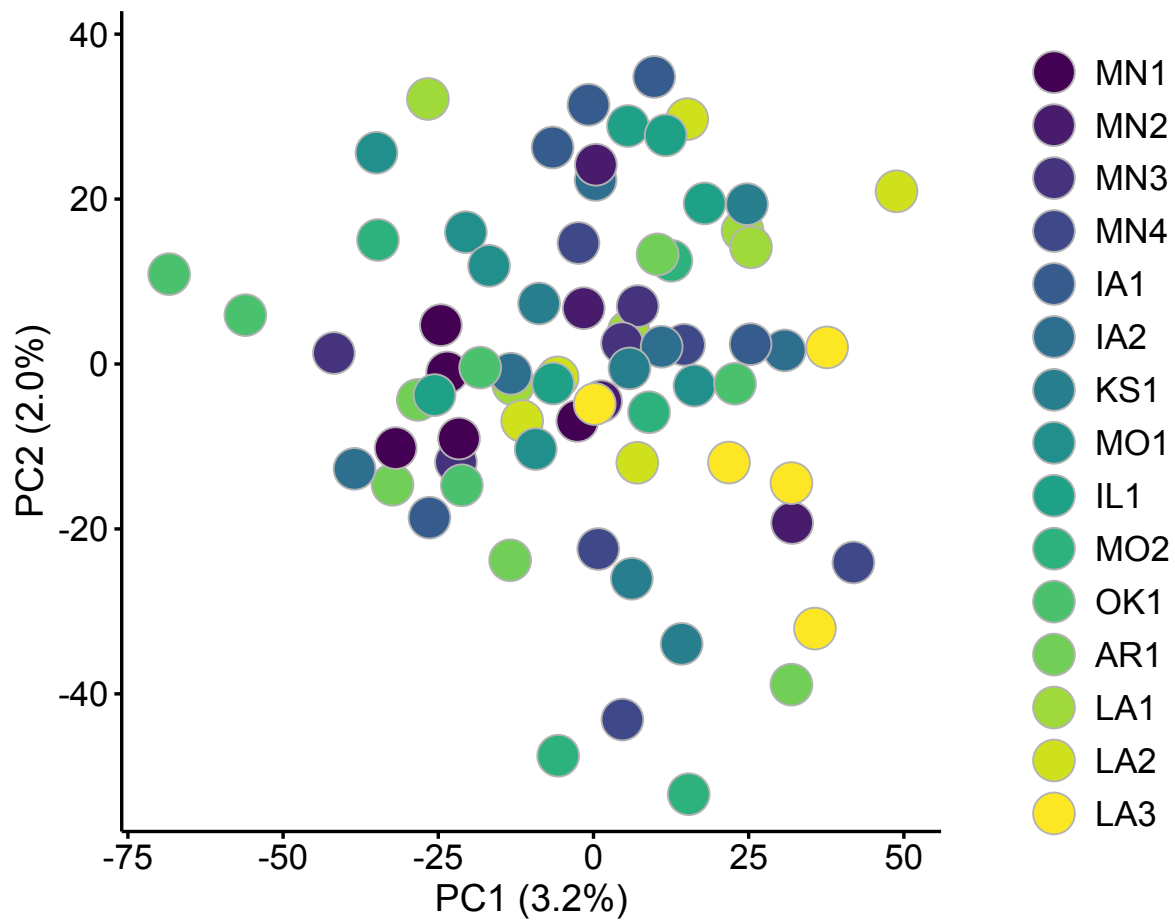

Supplement: S11 Fig — Reads were aligned to the A. artemisiifolia de novo transcriptome. (PDF) [file pgen.1008707.s011.pdf]

Uncorrected

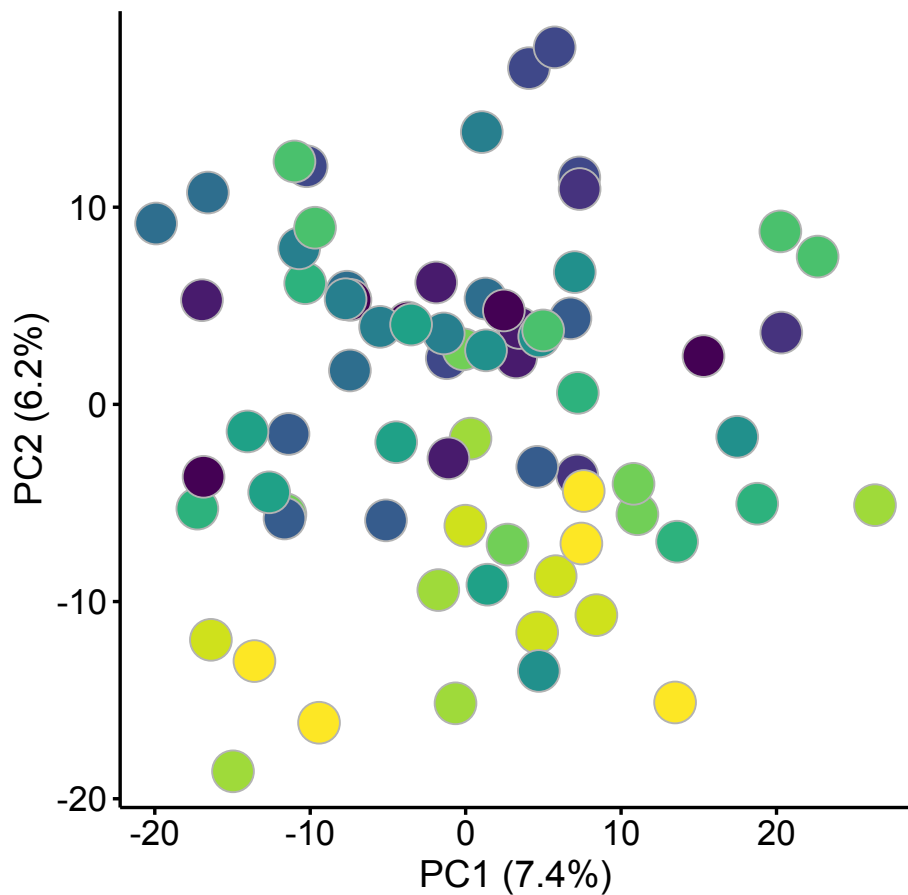

Corrected

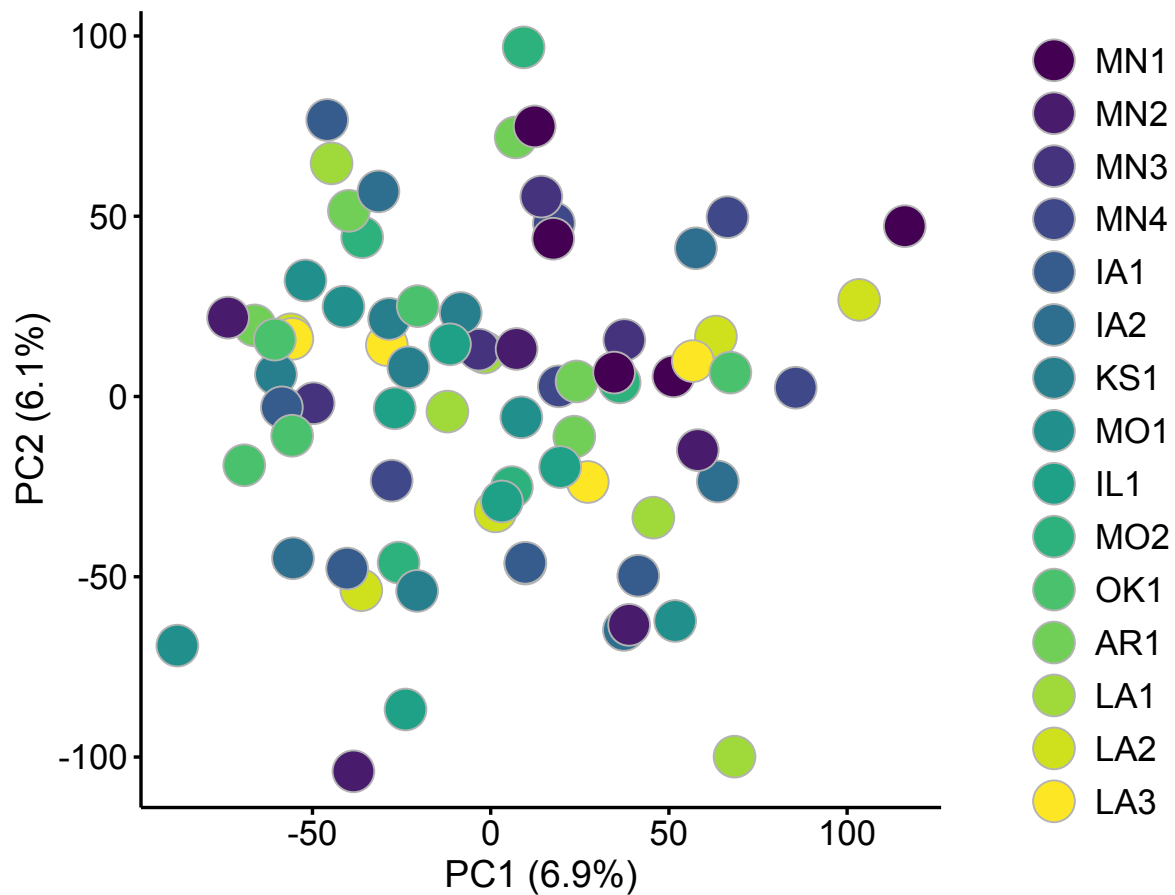

Supplement: S12 Fig — Reads were aligned to the H. annuus reference genome. (PDF) [file pgen.1008707.s012.pdf]

Optimum = 1

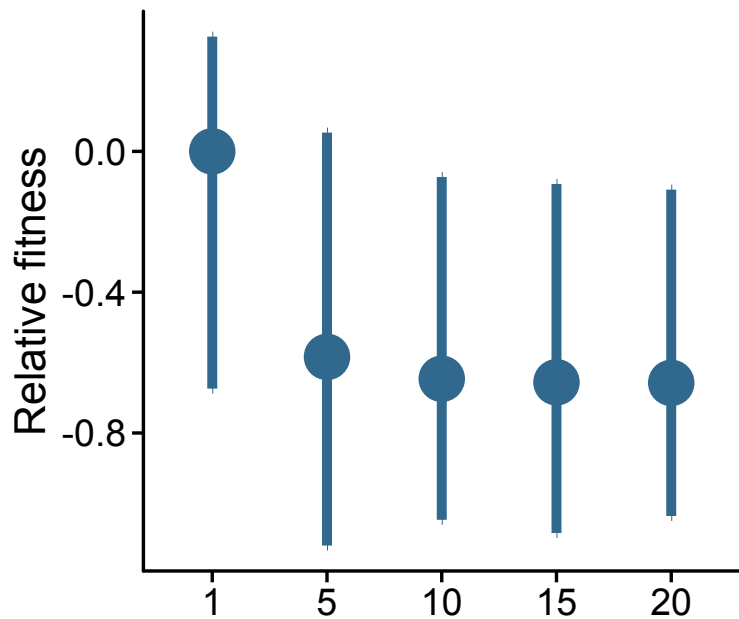

Optimum = 5

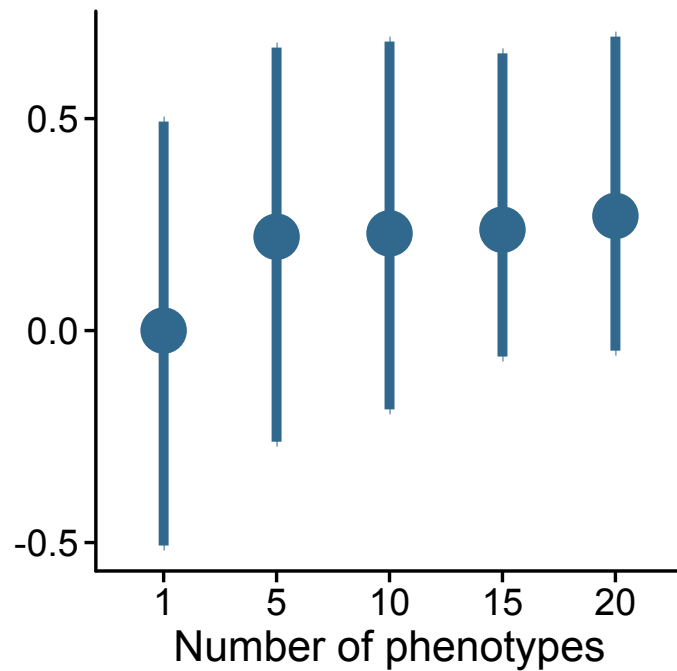

Optimum = 10

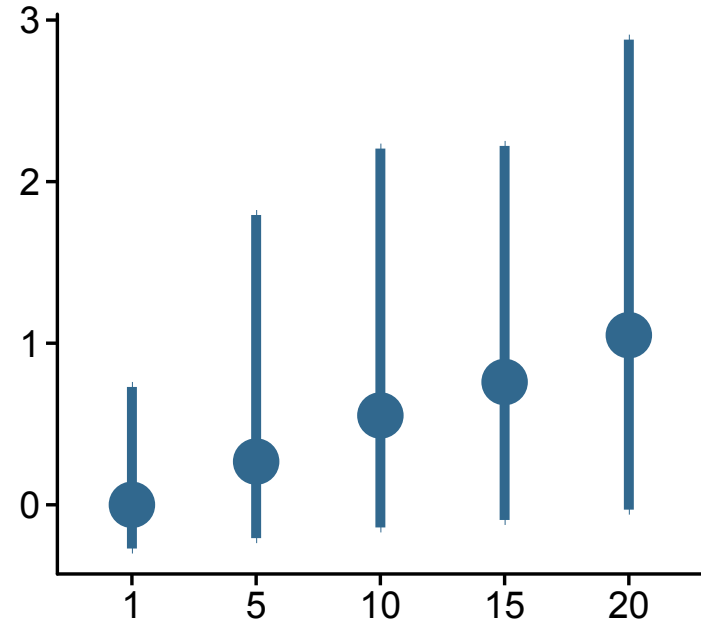

Supplement: S13 Fig — Phenotypes start from an initial value 0 and selection acts to move them towards three different optima. Shown are medians and interquartile ranges (IQR) from 300 simulations. The fitness estimates were normalized in relation to the non-pleiotropic class (median = 0, IQR = 1). (PDF) [file pgen.1008707.s013.pdf]
