# Supplementary material for: Pleiotropy facilitates local adaptation to distant optima in common ragweed (Ambrosia artemisiifolia)
Source: PLoS Genet. 2020 Mar 25;16(3):e1008707. doi: 10.1371/journal.pgen.1008707 (PMC7135370; doi:10.1371/journal.pgen.1008707)
Supplement: S1 Table — (PDF) [file pgen.1008707.s014.pdf]

| | MN1 | MN2 | MN3 | MN4 | IA1 | IA2 | KS1 | MO1 | IL1 | MO2 | OK1 | AR1 | LA1 | LA2 |
|---|---|---|---|---|---|---|---|---|---|---|---|---|---|---|
| MN2 | 0.07 | | | | | | | | | | | | | |
| MN3 | 0.07 | 0.07 | | | | | | | | | | | | |
| MN4 | 0.07 | 0.07 | 0.07 | | | | | | | | | | | |
| IA1 | 0.06 | 0.07 | 0.06 | 0.07 | | | | | | | | | | |
| IA2 | 0.07 | 0.07 | 0.08 | 0.07 | 0.07 | | | | | | | | | |
| KS1 | 0.08 | 0.08 | 0.08 | 0.08 | 0.07 | 0.06 | | | | | | | | |
| MO1 | 0.06 | 0.07 | 0.07 | 0.07 | 0.06 | 0.07 | 0.08 | | | | | | | |
| IL1 | 0.07 | 0.07 | 0.07 | 0.07 | 0.06 | 0.07 | 0.08 | 0.06 | | | | | | |
| MO2 | 0.06 | 0.07 | 0.06 | 0.07 | 0.06 | 0.07 | 0.07 | 0.06 | 0.06 | | | | | |
| OK1 | 0.07 | 0.08 | 0.08 | 0.08 | 0.07 | 0.07 | 0.07 | 0.07 | 0.08 | 0.07 | | | | |
| AR1 | 0.09 | 0.08 | 0.08 | 0.08 | 0.07 | 0.08 | 0.09 | 0.07 | 0.07 | 0.06 | 0.08 | | | |
| LA1 | 0.09 | 0.11 | 0.10 | 0.10 | 0.09 | 0.11 | 0.11 | 0.09 | 0.09 | 0.08 | 0.10 | 0.08 | | |
| LA2 | 0.09 | 0.10 | 0.09 | 0.09 | 0.09 | 0.10 | 0.11 | 0.08 | 0.09 | 0.08 | 0.10 | 0.08 | 0.08 | |
| LA3 | 0.11 | 0.12 | 0.12 | 0.12 | 0.11 | 0.13 | 0.13 | 0.11 | 0.11 | 0.10 | 0.12 | 0.10 | 0.11 | 0.09 |
