# Supplementary material for: Pleiotropy facilitates local adaptation to distant optima in common ragweed (Ambrosia artemisiifolia)
Source: PLoS Genet. 2020 Mar 25;16(3):e1008707. doi: 10.1371/journal.pgen.1008707 (PMC7135370; doi:10.1371/journal.pgen.1008707)
Supplement: S2 Table — (PDF) [file pgen.1008707.s015.pdf]

	θ_π	θ_w	Tajima's *D*
MN1	0.019	0.021	–0.732
MN2	0.019	0.020	–0.599
MN3	0.019	0.020	–0.752
MN4	0.019	0.021	–0.711
IA1	0.019	0.021	–0.738
IA2	0.019	0.021	–0.734
KS1	0.019	0.021	–0.649
MO1	0.019	0.021	–0.736
IL1	0.019	0.021	–0.701
MO2	0.019	0.021	–0.757
OK1	0.019	0.021	–0.723
AR1	0.019	0.020	–0.613
LA1	0.018	0.019	–0.454
LA2	0.018	0.020	–0.574
LA3	0.017	0.018	–0.302
