# Supplementary material for: Pleiotropy facilitates local adaptation to distant optima in common ragweed (Ambrosia artemisiifolia)
Source: PLoS Genet. 2020 Mar 25;16(3):e1008707. doi: 10.1371/journal.pgen.1008707 (PMC7135370; doi:10.1371/journal.pgen.1008707)
Supplement: S3 Table — (PDF) [file pgen.1008707.s016.pdf]

	PC1	PC2	PC3	PC4	PC5	PC6	PC7	PC8	PC9	PC10
BIO1	0.06	0.01	0.01	0.04	0.05	0	0.03	0.01	0	0.02
BIO2	0.01	0.2	0.06	0.11	0.12	0.1	0.02	0	0	0.05
BIO3	0.06	0.03	0	0.05	0.06	0.08	0.08	0.03	0.02	0.09
BIO4	0.06	0	0.01	0.01	0.02	0.06	0.06	0.07	0.05	0.05
BIO5	0.05	0.08	0.05	0.09	0.09	0.02	0.04	0.1	0.01	0.03
BIO6	0.06	0	0	0.03	0.04	0.02	0.04	0.01	0.03	0.01
BIO7	0.06	0.03	0.02	0	0.02	0.04	0.08	0.05	0.05	0.02
BIO8	0.03	0.15	0.11	0.2	0.07	0.06	0.05	0.04	0.06	0
BIO9	0.06	0.05	0.04	0.01	0.09	0.22	0.02	0.01	0.16	0.03
BIO10	0.06	0.03	0.03	0.07	0.09	0.06	0.04	0.1	0.03	0.03
BIO11	0.06	0.01	0	0.03	0.04	0.02	0.04	0.01	0.02	0.01
BIO12	0.06	0.01	0	0.04	0.03	0.03	0.01	0.05	0.06	0.08
BIO13	0.05	0.05	0.15	0.02	0.01	0.03	0.14	0.17	0.02	0.08
BIO14	0.06	0.06	0.06	0.07	0.06	0.03	0.09	0.06	0.06	0.06
BIO15	0.05	0.07	0.16	0.04	0.04	0.04	0.12	0.04	0.04	0.11
BIO16	0.06	0.05	0.12	0.05	0.04	0.04	0.03	0.03	0.18	0.12
BIO17	0.06	0.03	0.07	0.06	0.02	0.01	0.02	0.02	0.07	0.07
BIO18	0.04	0.14	0.04	0.02	0.07	0.13	0.04	0.18	0.12	0.02
BIO19	0.06	0	0.05	0.06	0.04	0.02	0.06	0.01	0.02	0.1
PVE	0.80	0.12	0.03	0.03	0.01	0.01	0	0	0	0

For explanations of the BIO-variables, see https://www.worldclim.org/bioclim
